# Combination treatment with highly bioavailable curcumin and NQO1 inhibitor exhibits potent antitumor effects on esophageal squamous cell carcinoma

**DOI:** 10.1007/s00535-019-01549-x

**Published:** 2019-02-08

**Authors:** Ayaka Mizumoto, Shinya Ohashi, Mayumi Kamada, Tomoki Saito, Yukie Nakai, Kiichiro Baba, Kenshiro Hirohashi, Yosuke Mitani, Osamu Kikuchi, Junichi Matsubara, Atsushi Yamada, Tsukasa Takahashi, Hyunjin Lee, Yasushi Okuno, Masashi Kanai, Manabu Muto

**Affiliations:** 1 Department of Therapeutic Oncology, Graduate School of Medicine, Kyoto University, 54 Kawaharacho, Shogoin, Sakyo-ku, Kyoto, 606-8507 Japan; 2 Department of Biomedical Data Intelligence, Graduate School of Medicine, Kyoto University, 53 Kawaharacho, Shogoin, Sakyo-ku, Kyoto, 606-8507 Japan; 3 Department of Medical Oncology, Dana-Farber Cancer Institute, 450 Brookline Ave, 866-408-DFCI (3324), Boston, MA 02215 USA; 4 Theravalues Corporation, 3-12 Kioicho, Chiyoda-ku, Tokyo, 102-0094 Japan

**Keywords:** Esophageal squamous cell carcinoma, Curcumin, Theracurmin^®^, NQO1, NQO1 inhibitor

## Abstract

**Background:**

Esophageal squamous cell carcinoma (ESCC) is one of the most intractable cancers, so the development of novel therapeutics has been required to improve patient outcomes. Curcumin, a polyphenol from Curcuma longa, exhibits various health benefits including antitumor effects, but its clinical utility is limited because of low bioavailability. Theracurmin^®^ (THC) is a highly bioavailable curcumin dispersed with colloidal submicron particles.

**Methods:**

We examined antitumor effects of THC on ESCC cells by cell viability assay, colony and spheroid formation assay, and xenograft models. To reveal its mechanisms, we investigated the levels of reactive oxygen species (ROS) and performed microarray gene expression analysis. According to those analyses, we focused on NQO1, which involved in the removal of ROS, and examined the effects of NQO1-knockdown or overexpression on THC treatment. Moreover, the therapeutic effect of THC and NQO1 inhibitor on ESCC patient-derived xenografts (PDX) was investigated.

**Results:**

THC caused cytotoxicity in ESCC cells, and suppressed the growth of xenografted tumors more efficiently than curcumin. THC increased ROS levels and activated the NRF2–NMRAL2P–NQO1 expressions. Inhibition of NQO1 in ESCC cells by shRNA or NQO1 inhibitor resulted in an increased sensitivity of cells to THC, whereas overexpression of NQO1 antagonized it. Notably, NQO1 inhibitor significantly enhanced the antitumor effects of THC in ESCC PDX tumors.

**Conclusions:**

These findings suggest the potential usefulness of THC and its combination with NQO1 inhibitor as a therapeutic option for ESCC.

**Electronic supplementary material:**

The online version of this article (10.1007/s00535-019-01549-x) contains supplementary material, which is available to authorized users.

## Introduction

Esophageal squamous cell carcinoma (ESCC) is the major histological type of esophageal cancer [1, 2], which is the sixth leading cause of cancer-related mortality and the eighth most common cancer worldwide [3, 4]. Despite recent progress in systematic therapeutics, ESCC remains one of the most intractable cancers, having an extremely low 5-year survival rate [5, 6]. Therefore, the development of novel treatment options has been needed to improve the outcomes for ESCC patients.

Curcumin is a naturally occurring polyphenol derived from the root of *Curcuma longa* that is recognized as a generally safe compound by the Food and Drug Administration [7, 8]. Curcumin demonstrates various biological benefits including antimicrobial and anti-inflammatory actions, and is involved in the regulation of programmed cell death and survival pathways by modulating transcription factors such as nuclear factor-κB, growth factors, inflammatory cytokines, and receptors [9]. Curcumin has been shown to have antitumor effects on several types of cancer cells including lung cancer [10], glioblastoma [11], colon cancer [12], pancreatic cancer [13], prostate cancer [14], and ESCC [15–17].

Despite the demonstration of the promising antitumor effects of curcumin in preclinical studies, its clinical use is currently limited because of its poor bioavailability in humans [18]. Curcumin is not easily soluble in water [19], and oral administration of curcumin does not achieve sufficient blood concentrations to exert therapeutic efficacy [20–22]. To overcome this limitation, various strategies of drug development have been attempted to improve the bioavailability of curcumin [23–27].

Theracurmin^®^ (THC, curcumin content 30% w/w) is an effective preparation of curcumin dispersed with colloidal submicron particles, making it easily disperse in water [22]. Consequently, the bioavailability of curcumin in THC is much improved, and the area under the blood concentration–time curve (AUC) after the oral administration of THC is more than 40-fold higher than that of curcumin in rats and 27-fold higher than that of curcumin in humans [22]. In fact, THC has been reported to be clinically useful for treating osteoarthritis [28], muscle damage [29], and atherosclerotic hyperlipidemia [30]. With regard to experimental cancer research, the cytotoxicity or antitumor effects of THC have been reported using several cancer cell lines [31, 32], but the effectiveness of THC against ESCC has not been fully clarified.

The purposes of our study were to investigate the antitumor effects of THC on ESCC cells and to compare the effects of curcumin and THC in vivo. Here, we found that induction of NAD(P)H quinone dehydrogenase 1 (NQO1), which is the enzyme that scavenge reactive oxygen species (ROS) [33], plays an antagonistic role in THC-induced antitumor effects, and we, therefore, examined the effects on ESCC of a combination treatment with THC and NQO1 inhibitor.

## Materials and methods

### In vitro assay and analysis

Methods for cell culture, WST-1 cell viability assay, Caspase-Glo^®^ 3/7 assay, spheroid assay, soft agar colony formation assay, microarray hybridization, real-time reverse transcription–polymerase chain reaction (RT–PCR), western blotting, chromatin protein isolation, measurement of intracellular and/or mitochondrial ROS levels, immunofluorescent staining for 8-hydroxy-2′-deoxyguanosine (8-OHdG), cell cycle assay, senescence-associated β-galactosidase (SABG) assay, viral infections, and metabolite analysis are described in Supplementary materials and methods.

### Assessment of bioavailability and antitumor effects of curcumin and THC in vivo

All animal experiments conformed to the relevant regulatory standards and were approved by the Institutional Animal Care and Use Committee of Kyoto University (Med Kyo 18284).

C57BL/6 male mice (CLEA Japan, Inc., Tokyo, Japan) were given either a control diet (without curcumin or THC), a curcumin diet (containing 0.6 g/kg curcumin), or a THC diet (containing 2 g/kg THC that included 0.6 g/kg curcumin). After 1 week, blood was taken from the heart of mice and placed into heparinized tubes. Plasma was immediately prepared by centrifugation at 1000*g*, 4 °C for 10 min and stored at − 80 °C until use. The plasma concentration of curcumin was measured using high-performance liquid chromatography–tandem mass spectrometry (MS)/MS as described previously [22].

To compare the tumor growth-inhibitory effects of curcumin and THC, xenografted tumors derived from TE-11R cells were used. TE-11R cells (1.5 × 10^6^ cells) were suspended in 50% Matrigel (BD Biosciences, San Jose, CA), followed by subcutaneous implantation into the left flank of 6-week-old hairless SCID male mice (Charles River Laboratories Japan Inc. Yokohama, Japan) (*n* = 15, day 0). The mice were randomly assigned to three groups (*n* = 5 each) and received either control, curcumin, or THC diet from day 0 to day 70.

The tumors were measured with a caliper, and tumor volume (mm^3^) was calculated using the following formula: (length) × (width)^2^ × 0.5.

### Assessment of antitumor effects of THC and NQO1 inhibitor in vivo

Patient-derived xenograft (PDX) ESCC tumors were utilized to assess the therapeutic effects of THC and NQO1 inhibitor in vivo. All experiments conformed to the relevant regulatory standards and were approved by the Institutional Animal Care and Use Committee of Kyoto University (Med Kyo 18284) and the Ethics Committee of Kyoto University (G0770).

To establish PDX tumors, biopsy specimens taken from human ESCC tissue of primary site were placed in a subcutaneous pocket created by a 5-mm incision in the left flank of 6-week-old hairless SCID male mice (*n* = 20), which was then closed by suturing. Mice were randomly assigned to one of the four groups at day 21 (*n* = 5 each), and given either normal water or THC-containing water (5000 ppm) for drinking from day 21 to day 70. Either DMSO (mock) or 5-methoxy-1,2-dimethyl-3-[(4-nitrophenoxy)methyl]indole-4,7-dione (ES936) (sc-362737, Santa Cruz Biotechnology, Inc., CA, USA) (5 mg/kg) was administered intraperitoneally every other day from day 21 to day 70.

The tumors were monitored with a caliper, and tumor volume (mm^3^) was calculated using the following formula: (length) × (width)^2^ × 0.5.

### Immunohistochemical staining

Immunohistochemical staining was performed as described previously [34]. Additional information is given in Supplementary materials and methods.

### Statistical analyses

Data are presented as the mean ± standard error (SE) of triplicate experiments unless otherwise stated. Differences between two groups were analyzed using the 2-tailed Student’s *t* test, and *P < 0.05 and **P < 0.01 were considered significant. All statistical analyses were performed using SPSS 21 for Windows (SPSS Inc., Chicago, IL, USA).

## Results

### Cytotoxic and antiproliferative effects of THC on ESCC cells

**Fig. 1:**
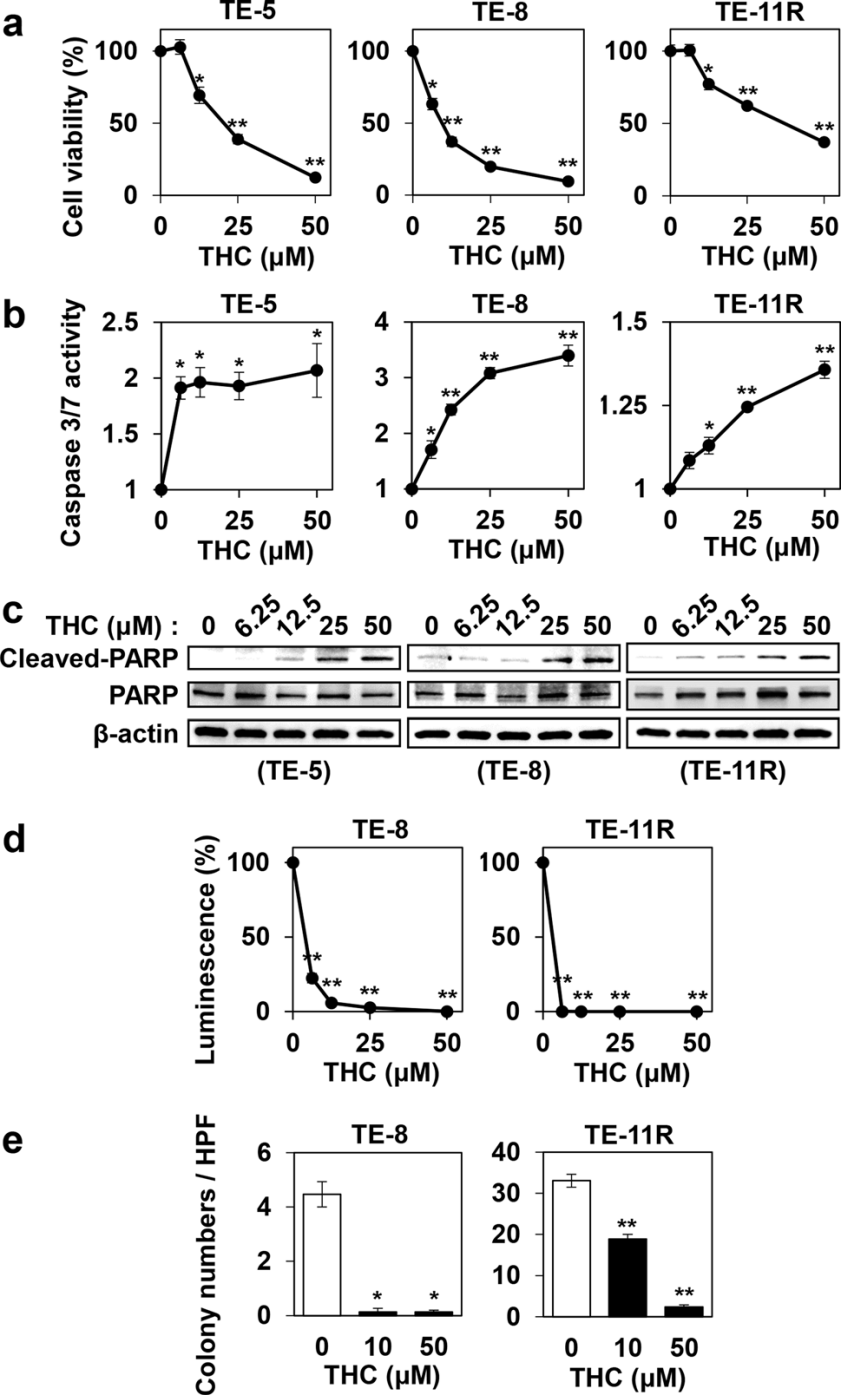
Cytotoxic effects of Theracurmin^®^ on ESCC cells. **a** Cell viability: ESCC cells were cultured with the indicated concentrations (0–50 μM) of Theracurmin^®^ (THC) for 96 h. Cell viability was measured using the WST-1 assay. Viability of each cell type treated with THC relative to that of untreated cells is indicated (*P < 0.05, **P < 0.01, vs untreated cells; *n* = 3). **b** Caspase 3/7 activity: ESCC cells were cultured with the indicated concentrations (0–50 μM) of THC for 12 h (TE-5) or 24 h (TE-8 and TE-11R). Caspase 3/7 activity was measured using the Caspase-Glo 3/7 Assay. Relative Caspase 3/7 activity of each cell type treated with the indicated concentrations of THC relative to that of untreated cells is indicated (*P < 0.05, **P < 0.01, vs untreated cells; *n* = 3). **c** Assessment of apoptosis: ESCC cells were cultured with the indicated concentrations (0–50 μM) of THC for 24 h. The cleavage of PARP was investigated by western blotting. β-actin served as a loading control. **d** Spheroid assay: Spheres were generated by TE-8 and TE-11R cells treated with the indicated concentrations (0–50 μM) of THC for 2 weeks. Spheroid formation level was measured using the CellTiter-Glo 3D Cell Viability Assay. Spheroid formation by each cell type treated with THC relative to that of untreated cells is indicated (**P < 0.01, vs untreated cells; *n* = 3). **e** Colony formation assay: Colonies were generated by TE-8 and TE-11R cells with the indicated concentrations (0–50 μM) of THC for 2 weeks (TE-11R) or 3 weeks (TE-8). Colony numbers per high-power field (HPF) were counted in 15 random fields to determine the mean colony-forming units for each sample (*P < 0.05, **P < 0.01, vs untreated cells)

We treated ESCC cells (TE-1, TE-5, TE-6, TE-8, TE-10, TE-11, TE-11R, T.Tn, and HCE-4 cells) with various concentrations of THC for 96 h, and then determined cell viability. As shown in Fig. 1a and Supplementary Fig. 1, THC dose-dependently decreased cell viability in all ESCC cells. The 50% inhibitory concentration (IC_50_) values of THC for each ESCC cell are shown in Supplementary Table 1. We selected 3 cell lines, TE-5, TE-8, and TE-11R cells, for subsequent experiments, because TE-5 cells are derived from poorly-differentiated ESCC [35], and TE-8 and TE-11R cells were suitable for spheroid and colony formation assays.

Next, we measured caspase 3 and caspase 7 activity in those cells treated with THC. Treatment with THC significantly increased the caspase activity as well as the cleavage of Poly (ADP-ribose) polymerase (PARP) protein, showing the capability of THC to induce apoptosis (Fig. 1b, c).

In addition, we investigated whether THC influences cell cycle and/or senescence in ESCC cells. As shown in Supplementary Fig. 2a, THC induced G2/M cell cycle arrest in ESCC cells. Moreover, THC significantly increased SABG-positive senescent cells in ESCC cells (Supplementary Fig. 2b).

Collectively, these results demonstrated the cytotoxic and antiproliferative effects of THC on ESCC cells.

### Inhibitory effects of THC for spheroid and colony formation in ESCC cells

We performed spheroid and soft agar colony formation assays to investigate the effect of THC on the stem cell-like properties and anchorage-independent cell growth activities of ESCC cells. THC showed a strong dose-dependent inhibition of spheroid formation (Fig. 1d) and colony formation (Fig. 1e) in both TE-8 and TE-11R cells.

### Bioavailability and antitumor effects of THC against ESCC xenografts in vivo

**Fig. 2:**
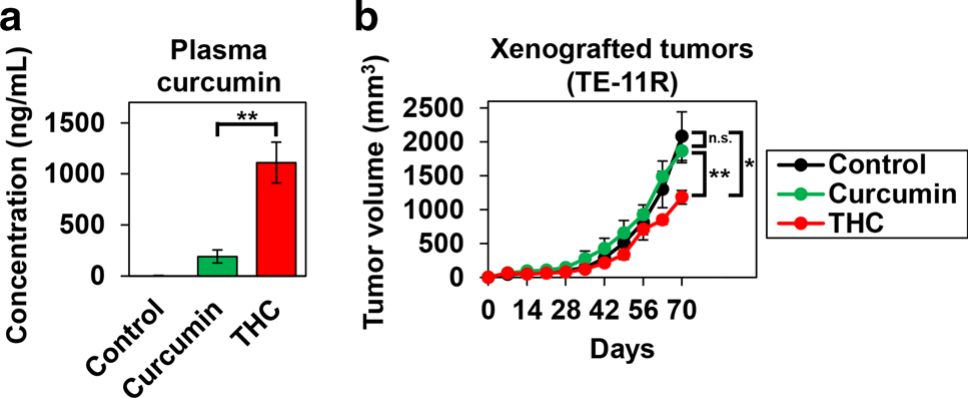
Comparison of blood curcumin concentration and antitumor effect on ESCC after ingestion of curcumin and Theracurmin^®^ diet. **a** Plasma concentration of curcumin: C57BL/6 mice received control, curcumin (0.6 g/kg) or Theracurmin^®^ (THC) (2 g/kg: containing 0.6 g/kg curcumin) diet for 1 week. Plasma concentration of curcumin was measured (**P < 0.01, vs curcumin diet; *n* = 5). **b** Xenografted-tumor growth: TE-11R cells (1.5 × 10^6^ cells) were injected into hairless SCID mice that received control, curcumin, or THC diet for 70 days. The tumor volume was measured with a caliper [*P < 0.05, **P < 0.01, n.s. (non significant, *P *> 0.05), vs control or curcumin diet; *n* = 5]

To examine the difference in bioavailability between curcumin and THC in vivo, we fed mice either a curcumin diet (curcumin group) or a THC diet (THC group) containing equal amounts of curcumin for a week. There was no significant difference in the dietary intake of the groups. In addition, there were no obvious abnormalities in their general condition (e.g., adverse hematological effects and body weight loss) (data not shown). We examined plasma curcumin concentrations in these mice, and the THC group showed markedly higher plasma levels of curcumin than the curcumin group (1110.4 ± 199.8 ng/mL vs 191.1 ± 64.4 ng/mL) (Fig. 2a). To investigate whether this better bioavailability of THC led to a stronger antitumor effect in vivo, we compared the tumor growth inhibition in ESCC (TE-11R) xenograft mice treated with THC or curcumin. As shown in Fig. 2b, the THC group demonstrated significantly greater tumor growth inhibition than the curcumin group (43% vs 11% at day 70).

### Upregulation of NMRAL2P after THC treatment

**Fig. 3:**
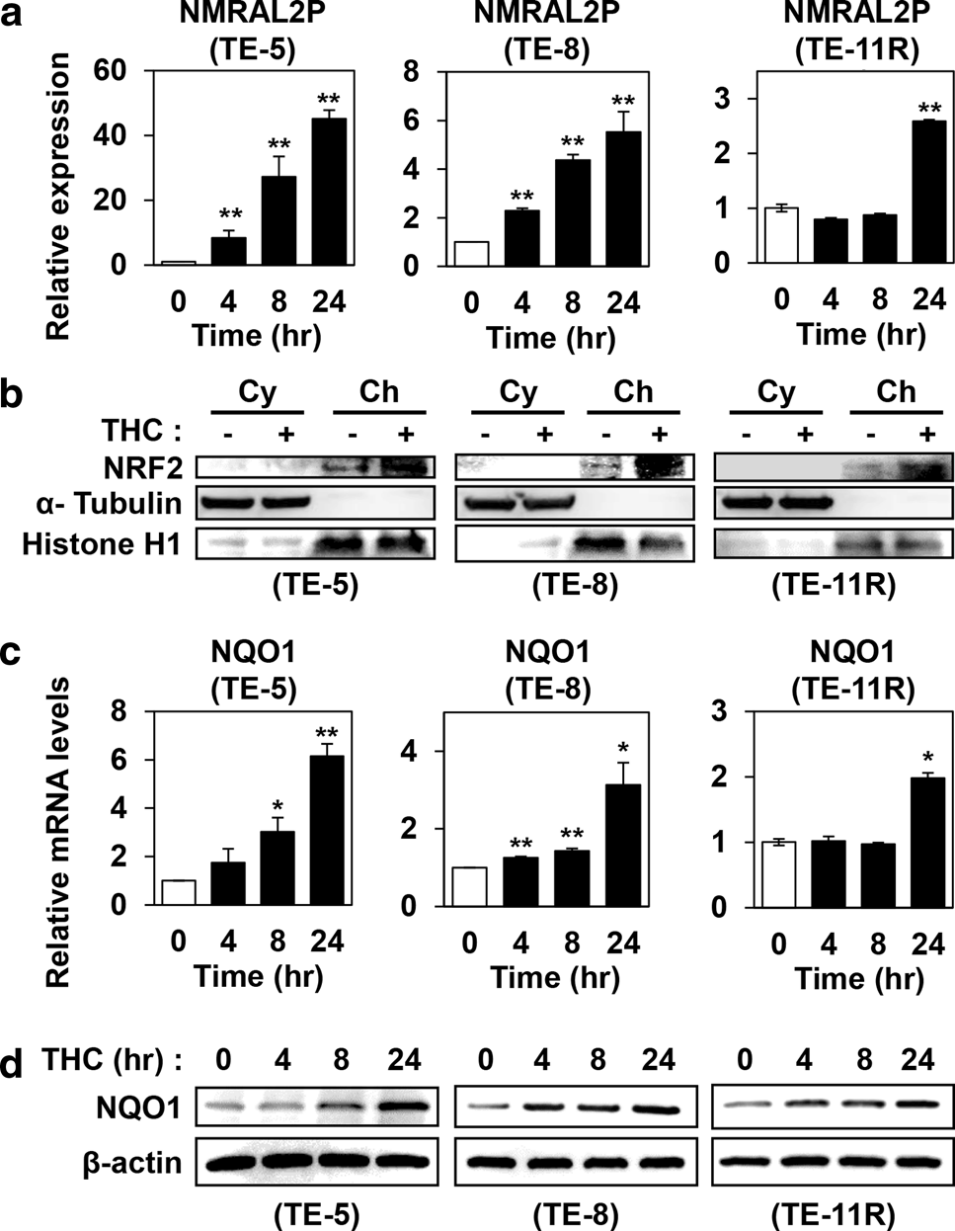
Activation of NRF2, and induction of NMRAL2P and NQO1 in ESCC cells treated with Theracurmin^®^. **a** NMRAL2P expression levels: ESCC cells were cultured with Theracurmin^®^ (THC, 10 μM) for the indicated times. NMRAL2P expression level was measured by RT–PCR. Relative NMRAL2P RNA levels of each cell type treated with THC for the indicated time relative to that of untreated cells are indicated. β-actin served as an internal control (**P < 0.01, vs untreated cells; *n* = 3). **b** NRF2 activation: ESCC cells were cultured with THC (TE-5: 10 μM, TE-8 and TE-11R: 50 μM) for 24 h. Cellular protein was divided into cytosolic fraction (Cy) and chromatin fraction (Ch). Western blotting was performed to reveal NRF2 activity. α-Tubulin or histone H1 served as a loading control for cytosolic or chromatin extracts, respectively. **c** NQO1 mRNA levels: ESCC cells were cultured with THC (10 μM) for the indicated times. NQO1 mRNA level was measured by RT–PCR. Relative NMRAL2P RNA levels of each cell type treated with THC for the indicated time relative to that of untreated cells are indicated. β-actin served as an internal control (*P < 0.05, **P < 0.01, vs untreated cells; *n* = 3). **d** NQO1 protein levels: ESCC cells were cultured with THC (TE-5: 10 μM; TE-8 and TE-11R: 50 μM) for the indicated times. NQO1 protein level was measured by western blotting. β-actin served as a loading control

Next, we performed microarray gene expression analysis to identify the changes in gene expression patterns in ESCC cells (TE-5 and TE-8 cells) after THC treatment. We identified several genes that showed differential RNA expression after THC treatment. Among these, expression levels of NmrA-like redox sensor 2 pseudogene (NMRAL2P, also known as Loc344887) were elevated in THC-treated TE-5 and TE-8 cells (log2 fold change: TE-5, 4 h; 2.95, 8 h; 4.35, 24 h; 4.77: TE-8, 8 h; 2.17, 24 h; 2.21, respectively) (Supplementary Fig. 3). We confirmed by RT–PCR that THC upregulated NMRAL2P expression time-dependently in TE-5, TE-8, and TE-11R cells (Fig. 3a).

### Activation of NRF2–NMRAL2P–NQO1 pathway by THC

Because NMRAL2P has been shown to be involved in the nuclear factor erythroid 2 like 2 (NRF2) pathway, in which NRF2 regulates NMRAL2P expression and NMRAL2P subsequently regulates expression of NQO1 [36–38], we next evaluated whether THC affected NRF2 activation and NQO1 expression in ESCC cells. As shown in Fig. 3b, THC increased nuclear, but not cytosolic, NRF2 protein levels, suggesting that THC treatment activated the NRF2 transcription factor. THC also increased mRNA and protein levels of NQO1 (Fig. 3c, d).

Kelch-like ECH-associated protein 1 (KEAP1) is a repressive partner of NRF2 that suppresses activation of the NRF2 pathway [39]. We next performed siRNA-mediated inhibition of KEAP1, NRF2, and NMRAL2P in TE-5 and TE-8 cells, and confirmed the reduced expression levels of the respective genes (Supplementary Fig. 4a–d). Knockdown of *KEAP1* resulted in an increased expression of NMRAL2P, while knockdown of *NRF2* caused a reduction of NMRAL2P levels in both TE-5 and TE-8 cells with and without THC treatment (Supplementary Fig. 4e). As shown in Supplementary Fig. 4f, g, knockdown of *NMRAL2P* or *NRF2* attenuated NQO1 mRNA and protein induction by THC. These results suggested that THC promoted the NRF2–NMRAL2P–NQO1 pathway via the activation of NRF2 transcription factor.

In addition, we examined the basal KEAP1 mRNA expression level or nuclear NRF2 protein level in ESCC cells, and we assessed the correlation between their expression levels and their differentiation status, morphological phenotype, and sensitivity to THC (Supplementary Tables 1, 2 and Supplementary Fig. 5a, b). Although nuclear NRF2 expression level is considered to be associated with the sensitivity to chemoradiotherapy in ESCC [40] and the expression of NRF2/KEAP1 might be associated with oncogenic characteristics in ESCC cells, there were no significant correlations between them in this study (data not shown).

### Increased intracellular and mitochondrial ROS levels induced by THC

**Fig. 4:**
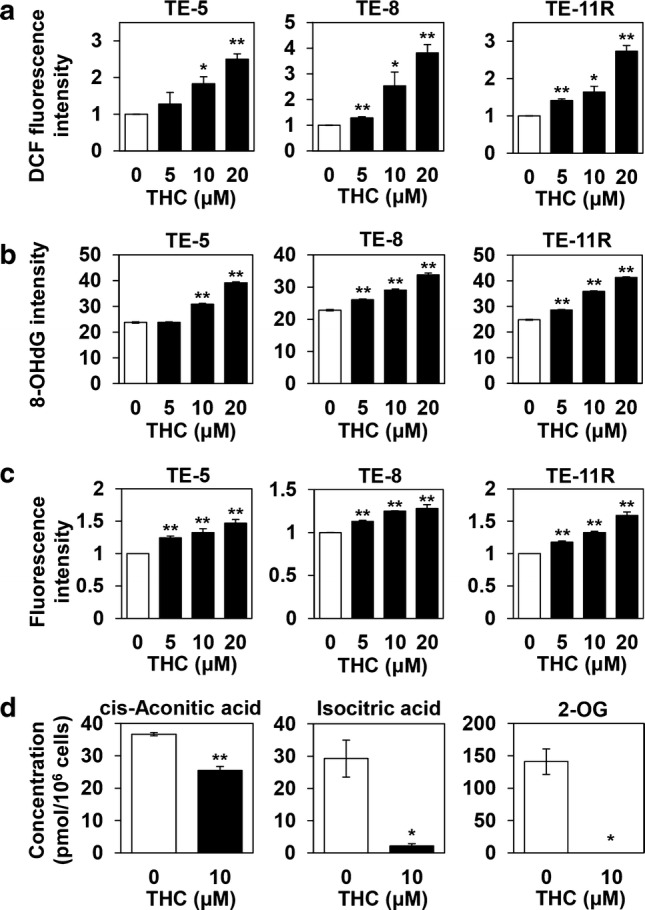
Theracurmin^®^-mediated oxidative damage in ESCC cells. **a** ROS production: ESCC cells were cultured with the indicated concentrations (0–20 μM) of Theracurmin^®^ (THC) for 1 h. Intracellular ROS levels were measured using the DCF assay. Relative DCF fluorescence intensity of each cell treated with the indicated concentrations of THC relative to that of untreated cells is indicated (*P < 0.05, **P < 0.01, vs untreated cells; *n* = 3). **b** Oxidative DNA damage: ESCC cells were cultured with the indicated concentrations (0–20 μM) of THC for 24 h. Oxidative DNA damage was measured by the intensity of immunofluorescent staining of 8-OHdG (**P < 0.01, vs untreated cells; *n* = 3). **c** Mitochondrial ROS levels: ESCC cells were cultured with the indicated concentrations (0–20 μM) of THC for 1 h. Mitochondrial ROS level was measured. Relative fluorescence intensity of each cell type treated with the indicated concentrations of THC relative to that of untreated cells is indicated (**P < 0.01, vs untreated cells; *n* = 3). **d** Metabolite analysis: TE-5 cells were cultured with THC (10 μM) for 8 h. The amounts of cis-aconitic acid, isocitric acid, and 2-OG were measured (*P < 0.05, **P < 0.01, vs untreated cells; *n* = 3)

Because NRF2 plays an important role in the response to oxidative stress [39], we measured the ROS levels in TE-5, TE-8, and TE-11R cells after THC treatment. As shown in Fig. 4a, THC significantly increased intracellular ROS levels in a dose-dependent manner. In addition, 8-OHdG, a marker of oxidative DNA damage, was increased by THC treatment (Fig. 4b), and mitochondrial ROS levels also showed a dose-dependent elevation after THC treatment (Fig. 4c).

### Inhibitory effect of THC on the TCA cycle

Because THC increased mitochondrial ROS levels in ESCC cells, we investigated whether THC affected mitochondrial functions such as the tricarboxylic acid (TCA) cycle. Metabolome analyses revealed that THC sharply decreased the levels of cis-aconitic acid, isocitric acid, and 2-oxoglutaric acid, components of the TCA cycle, indicating the inhibitory effects of THC on the TCA cycle (Fig. 4d).

### The role of NQO1 in ESCC cells with THC treatment

**Fig. 5:**
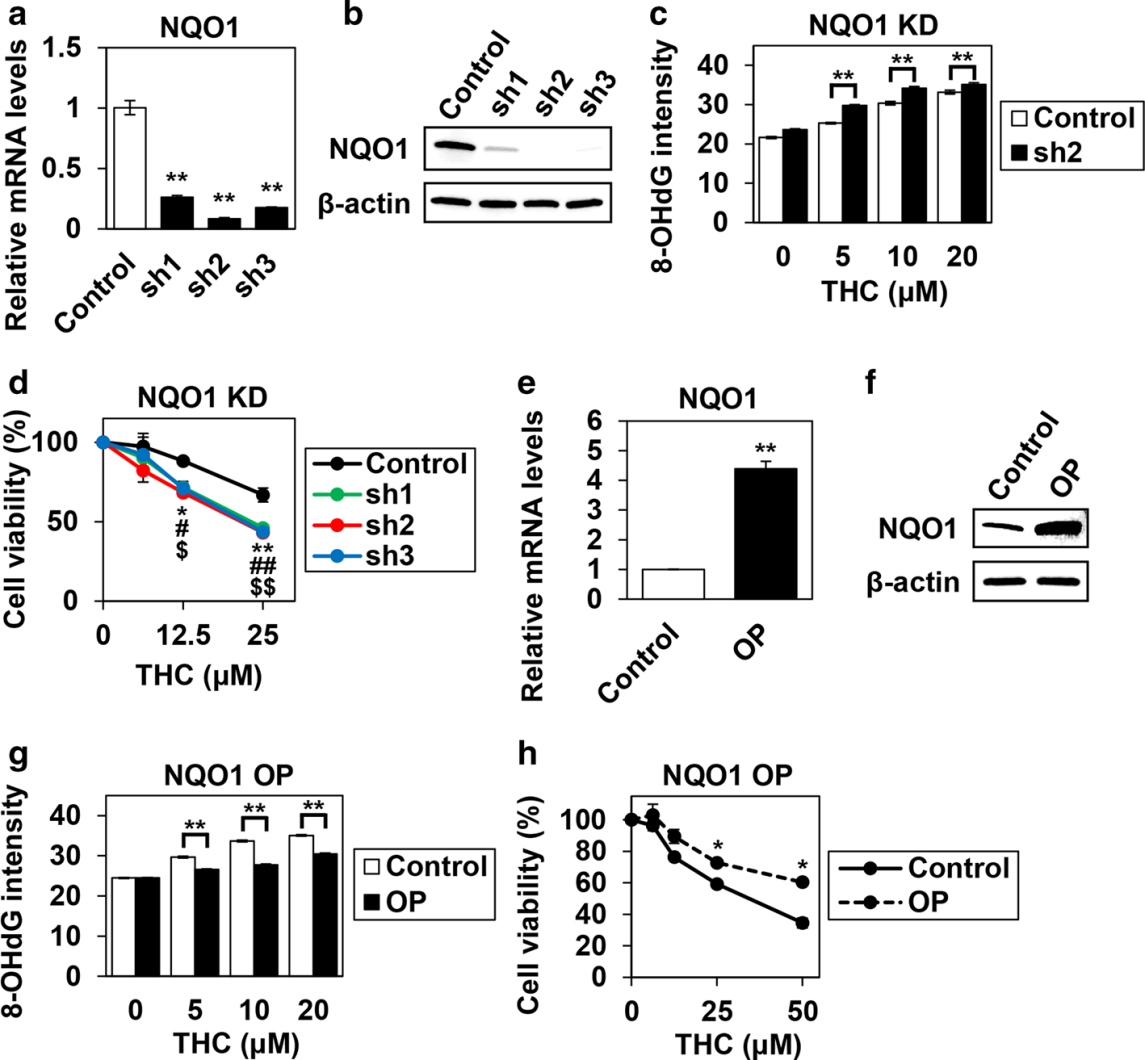
The role of NQO1 in Theracurmin^®^-treated ESCC cells. **a** NQO1 mRNA levels in *NQO1* knockdown cells: RT–PCR showed efficient *NQO1* knockdown using shRNA in TE-11R cells. Relative NQO1 mRNA level of each cell type relative to that of control cells is indicated. β-actin served as an internal control (**P < 0.01, vs the control cells; *n* = 3). **b** NQO1 protein levels in *NQO1* knockdown cells: Western blotting showed efficient *NQO1* knockdown using shRNA in TE-11R cells. β-actin served as a loading control. **c** Oxidative DNA damage in *NQO1* knockdown cells: *NQO1* knockdown cells were cultured with the indicated concentrations (0–20 μM) of THC for 24 h. Oxidative DNA damage was measured by the intensity of immunofluorescent staining for 8-OHdG (**P < 0.01, vs untreated cells). **d** Cell viability in *NQO1* knockdown cells: *NQO1* knockdown cells were cultured with the indicated concentrations of THC (0–25 μM) for 96 h. Cell viability was measured using the WST-1 assay. Viability of each cell type treated with the indicated concentrations of THC relative to that of untreated cells is indicated (sh1, *P < 0.05, **P < 0.01; sh2, ^#^*P *< 0.05, ^##^*P *< 0.01; sh3, ^$^*P *< 0.05, ^$$^*P *< 0.01; vs untreated control cells; *n* = 3) (**e**) NQO1 mRNA levels in NQO1-overexpressing cells: RT–PCR showed efficient NQO1 overexpression in TE-11R cells. NQO1 mRNA level of each cell type relative to that of control cells is indicated. β-actin served as an internal control (**P  < 0.01, vs control cells; *n *= 3) (**f**) NQO1 protein levels in NQO1-overexpressing cells: Western blotting showed efficient NQO1 overexpression in TE-11R cells. β-actin served as a loading control. **g** Oxidative DNA damage in NQO1-overexpressing cells: NQO1-overexpressing cells were cultured with the indicated concentrations (0–20 μM) of THC for 24 h. Oxidative DNA damage was measured by the intensity of immunofluorescent staining for 8-OHdG (**P < 0.01, vs untreated cells). **h** Cell viability in NQO1-overexpressing cells: NQO1-overexpressing cells were cultured with the indicated concentrations of THC (0–50 μM) for 96 h. Cell viability was measured using the WST-1 assay. Viability of each cell type treated with the indicated concentrations of THC relative to that of untreated cells is indicated (*P < 0.05, vs untreated control cells; *n* = 3) sh1-3: *NQO1* knockdown cells using shRNA 1-3, OP: NQO1-overexpressing cells

To investigate how NQO1 influences the cytotoxic effect of THC on ESCC cells, we created *NQO1* knockdown TE-11R cells using three types of shRNAs. As shown in Fig. 5a, b, NQO1 expression levels were remarkably reduced by all shRNAs at both mRNA and protein levels. *NQO1* knockdown resulted in an increase of 8-OHdG-indicated oxidative damage in THC-treated cells (Fig. 5c). Although *NQO1* knockdown alone did not affect cell growth (data not shown), susceptibility to THC in *NQO1* knockdown cells was significantly higher than that in control cells (Fig. 5d). Conversely, when we overexpressed NQO1 in TE-11R cells via lentivirus infection (Fig. 5e, f), NQO1 overexpression resulted in a decrease of 8-OHdG-indicated oxidative damage after THC treatment (Fig. 5g) and was associated with resistance to THC treatment (Fig. 5h).

### Combination effects of THC and NQO1 inhibitor in vitro

**Fig. 6:**
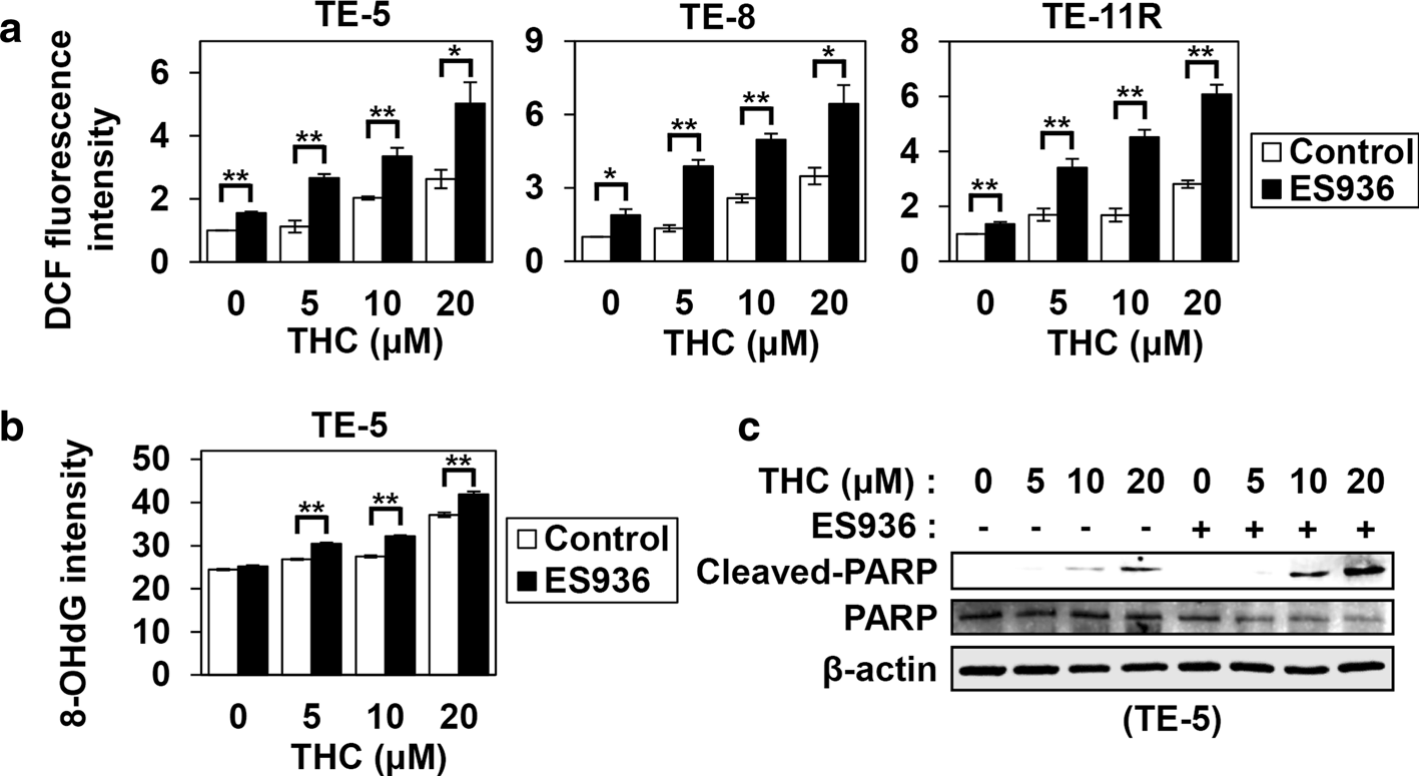
Combination effects of Theracurmin^®^ and NQO1 inhibitor in vitro. **a** ROS production in ESCC cells treated with both Theracurmin^®^ (THC) and NQO1 inhibitor: ESCC cells were cultured with the indicated concentrations (0–20 μM) of THC and ES936 (50 nM) for 1 h. ROS level was measured using the DCF assay. DCF fluorescence intensity of each cell type treated with THC and ES936 relative to that of untreated cells is indicated (*P < 0.05, **P < 0.01, vs the cells untreated with ES936; *n* = 3). **b** Oxidative DNA damage in TE-5 cells treated with both THC and NQO1 inhibitor: ESCC cells were cultured with the indicated concentrations (0–20 μM) of THC and ES936 (50 nM) for 24 h. Oxidative DNA damage was measured by the intensity of immunofluorescent staining for 8-OHdG (**P < 0.01, vs the cells untreated with ES936; *n* = approx. 350). **c** Assessment of apoptosis: TE-5 cells were cultured with the indicated concentrations (0–20 μM) of THC and ES936 (50 nM) for 24 h. The cleavage of PARP was investigated by western blotting. β-actin served as a loading control

Because of the additive effects of THC and NQO1 inhibition, we next examined whether NQO1 inhibitor enhanced the cytotoxic effects of THC. ESCC cells were treated with THC and ES936, which is a mechanism-based inhibitor of NQO1 [41]. ES936 has been shown not to decrease NQO1 protein expression [42]. The combination of THC and NQO1 inhibitor resulted in an increase in ROS production, 8-OHdG-indicated oxidative damage, and the cleavage of PARP protein (Fig. 6).

### Combination effect of THC and NQO1 inhibitor against ESCC PDX tumors in vivo

**Fig. 7:**
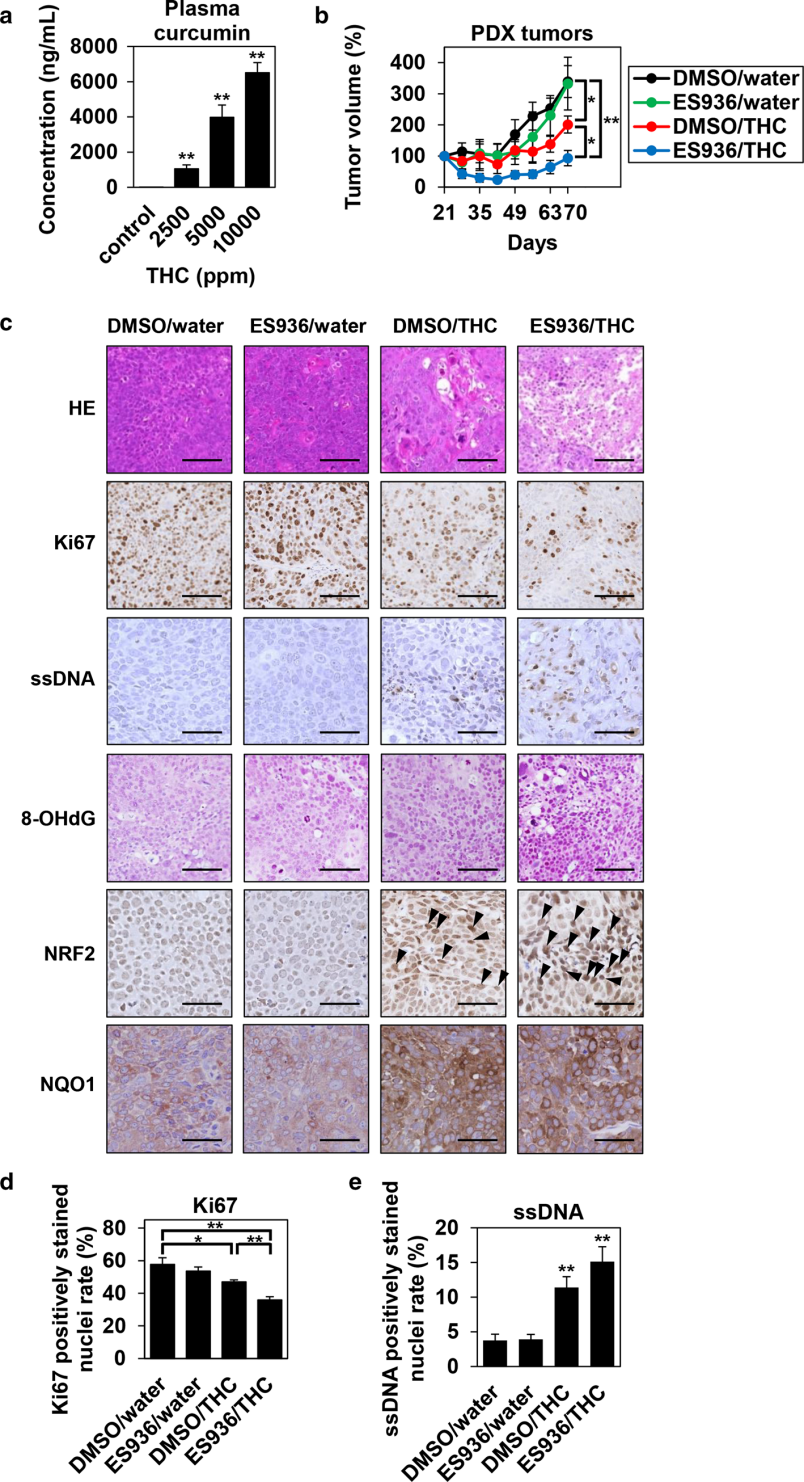
Antitumor effect of Theracurmin^®^ and NQO1 inhibitor against ESCC PDX tumors. **a** Plasma concentration of curcumin after ingesting Theracurmin^®^ (THC)-containing water: C57BL/6 mice received water-containing 2500, 5000, or 10000 ppm THC for 2 days. Plasma concentration of curcumin was measured (**P < 0.01, vs control; *n* = 5). **b** PDX tumor growth: Growth kinetics of subcutaneous ESCC PDX tumors treated with or without THC and/or NQO1 inhibitor (*P < 0.05, **P < 0.01, vs DMSO/water or DMSO/THC; *n* = 5). **c** Hematoxylin and eosin (HE) and immunohistochemical staining: HE and immunohistochemical staining for Ki67, ssDNA, 8-OHdG, NRF2, and NQO1 in ESCC PDX tumors treated with or without THC and/or NQO1 inhibitor. Scale bar = 100 μm (HE, Ki67, and 8-OHdG) and 50 μm (ssDNA, NRF2, and NQO1). Arrowheads indicate nuclear NRF2-positive cells. **d** Ki67 positively stained nuclei rate: Ki67 positively stained nuclei rate were counted in 6 random fields (*P < 0.05, **P < 0.01, vs DMSO/water or DMSO/THC). **e** ssDNA positively stained nuclei rate: ssDNA positively stained nuclei rate were counted in 6 random fields (*P < 0.05, **P < 0.01, vs DMSO/water)

To assess the antitumor effects of THC and NQO1 inhibitor in vivo, we evaluated the tumor growth inhibition in ESCC PDX tumors. First, to determine the optimal dose of THC, mice were given either normal water or THC-containing water (2500, 5000, or 10000 ppm), and plasma curcumin concentrations were examined. As shown in Fig. 7a, plasma curcumin levels increased THC dose-dependently. When 5000 ppm of THC was administered, the plasma concentration of curcumin reached 3973.8 ng/mL (about 11 μM), which was similar to the concentration we used in in vitro experiments. Therefore, we used water containing 5000 ppm THC for subsequent treatments. As shown in Fig. 7b, treatment with the combination of THC and NQO1 inhibitor resulted in a significant inhibition of PDX tumor growth compared with that after control or monotherapy (inhibition at day 70: NQO1 inhibitor 1.9%, THC 40.7%, THC plus NQO1 inhibitor 72.6%). There was no significant difference in the water intake between groups, and no significant adverse hematological effects or weight loss were detected (data not shown). Combination treatment with THC and NQO1 inhibitor significantly decreased Ki67 expression, a marker of cellular proliferation, compared with vehicle control and/or monotherapy with THC (Fig. 7c, d). In addition, combination treatment with THC and NQO1 inhibitor and/or THC monotherapy significantly increased single-stranded DNA (ssDNA), a marker of apoptosis, compared with vehicle control (Fig. 7c, e). Moreover, 8-OHdG, nuclear NRF2, and NQO1 levels were increased by treatment with THC alone as well as combination treatment with THC and NQO1 inhibitor (Fig. 7c). As a note, THC treatment did not cause any histological damages in normal esophageal tissues, and it did not increase ROS levels (8-OHdG levels) in normal esophageal tissues (data not shown).

## Discussion

Highly bioavailable curcumin (Theracurmin^®^, THC) showed antitumor effects on various types of ESCC cells and xenografted tumors. THC increased ROS levels in accompany with the activation of NRF2–NMRAL2P–NQO1 pathway. Since NQO1 revealed to play an antagonistic and antioxidative role in THC-induced cytotoxicity, we proposed a combination treatment with THC and NQO1 inhibitor. As a result, such a treatment strategy exhibited potent antitumor effects on ESCC PDX tumors.

In this study, biological effects (e.g., cytotoxic effects, ROS production, and sensitization of NQO1 inhibitor) of THC were similar to those of curcumin in vitro (Figs. 1a, 4a, 6c and Supplementary Fig. 6). We suggest that this is because THC is identical with curcumin as a component [22]. However, the antitumor effect of THC in vivo was much higher than that of an equal dose of curcumin. We suggest that the difference in the antitumor effect between THC and curcumin in vivo is caused by their different bioavailability (Fig. 2a).

The peak plasma curcumin levels following oral THC administration (5000 ppm) in mice reached 3973.8 ng/mL (Fig. 7a), which is roughly equivalent to 11 μM. In our in vitro experiments, we used 5–50 μM THC and the IC_50_ values of THC in various ESCC cells were between 7.03 and 34.98 μM. Because the plasma curcumin concentration after THC administration (400 mg curcumin/day) in humans was reported to be at about 3.7 μM [43], we presume that the dose of THC used in our experiments does not greatly exceed the achievable physiological range.

In the present study, NMRAL2P was upregulated in ESCC cells treated with THC. NMRAL2P is a long noncoding RNA that acts as a coactivator of NQO1 [38]. Although its precise function remains to be clarified, potential roles for NMRAL2P as a prognostic factor and/or a therapeutic target for cancer have been reported [44, 45]. Our results showed that *KEAP1* knockdown increased expression of NMRAL2P, while *NRF2* knockdown reduced it. Moreover, *NMRAL2P* knockdown inhibited NQO1 induction by THC. These results are consistent with the previous reports [36–38], and indicate that NMRAL2P is acting downstream of NRF2 and upstream of NQO1 in the NRF2-related signal cascade.

We showed that THC treatment increased intracellular and mitochondrial ROS levels in ESCC cells. These results were consistent with the previous reports showing curcumin-mediated ROS generation [46–48] that led to mitochondrial damage [49]. In this study, we also demonstrated that THC treatment affected the TCA cycle, which may retain a role in cancer cell metabolism. In particular, 2-oxoglutaric acid plays critical roles as a precursor of glutamine formation, as a nitrogen transporter for the urea cycle and/or ammonia detoxification, and as a cosubstrate for dioxygenases [50]. In addition, 2-oxoglutaric acid-dependent dioxygenases mediate the demethylation of DNA and histones, which is involved in regulation of the expression of many genes [51, 52], and depletion of 2-oxoglutaric acid was able to cause epigenetic changes [53]. Thus, the decrease in 2-oxoglutaric acid caused by THC may induce a range of aberrations in cellular metabolism.

We showed that THC increased nuclear NRF2 as well as NQO1 expressions. As NRF2 is upregulated by the ROS production [39] and NQO1 is a downstream factor of NRF2 [39], we suggest that nuclear NRF2 is increased via THC-mediated ROS production and NQO1 is upregulated via NRF2 activation. To examine the role of NQO1 in THC-mediated cytotoxicity in ESCC cells, we performed the experiments using cells with *NQO1* gene modification and/or NQO1 inhibitors. Knockdown of *NQO1* or administration of NQO1 inhibitor resulted in an increase of ROS and/or 8-OHdG levels in THC-treated ESCC cells, while overexpression of NQO1 resulted in a decrease of 8-OHdG in THC-treated ESCC cells. These results suggest that NQO1 plays an antioxidative role in THC-mediated cytotoxicity. As NQO1 acts as a reductase [33, 54–56], cells undergoing oxidative stress are considered to induce NQO1 to protect cells from those stresses. Accordingly, we thought that inhibition of NQO1 could enhance the antitumor effects of THC and we revealed that the strategy is effective.

We showed that basal levels of NQO1 protein in ESCC cells tended to be high, compared with those in normal esophageal epithelial cells (Supplementary Fig. 5c). Therefore, cytotoxic effect of THC may occur efficiently on ESCC cells in the presence of NQO1 inhibitor. As NQO1 inhibitor has not been used clinically, future studies to develop NQO1 inhibitors are warranted.

A limitation of this study is that we could not determine whether NMRAL2P regulates NQO1 directly or indirectly. Moreover, it remains unclear whether the antitumor effect of THC and NQO1 inhibitor is due to the direct effect on tumor cells or indirect effect on microenvironment. Further study will be required to address these questions.

In conclusion, THC exhibits in vitro and in vivo antitumor effects, and showed remarkably higher bioavailability and stronger antitumor effects than curcumin in vivo. THC induced ROS in accompany with the activation of NRF2–NMRAL2P–NQO1 pathway. NQO1 played an antioxidative role in THC-mediated cytotoxicity. Importantly, NQO1 inhibitor enhanced the THC-induced antitumor effects (Supplementary Fig. 7). These results suggest the potential usefulness of combination therapy with THC and NQO1 inhibitor for the treatment of ESCC.

## Electronic supplementary material

Below is the link to the electronic supplementary material.

Supplementary material 1 (DOCX 26 kb)

Supplementary material 2 (DOCX 14 kb)

Supplementary material 3 (DOCX 14 kb)

Supplementary material 4 (DOCX 20 kb)

Supplementary material 5 (TIFF 1933 kb)

Supplementary material 6 (TIFF 2282 kb)

Supplementary material 7 (TIFF 936 kb)

Supplementary material 8 (TIFF 571 kb)

Supplementary material 9 (TIFF 1715 kb)

Supplementary material 10 (TIFF 2676 kb)

Supplementary material 11 (TIFF 138 kb)

## Abbreviations

AUC: Area under the blood concentration–time curve
DCF: 2′,7′-dichlorodihydrofluorescein
DCFH-DA: 2′,7′-dichlorodihydrofluorescein diacetate
ESCC: Esophageal squamous cell carcinoma
FU: Fluorouracil
IC_50_: 50% inhibitory concentration
KEAP1: Kelch-like ECH-associated protein 1
MS: Mass spectrometry
NMRAL2P: NmrA-like redox sensor 2 pseudogene
NQO1: NAD(P)H quinone dehydrogenase
NRF2: Nuclear factor erythroid 2-like 2
PARP: Poly (ADP-ribose) polymerase
PBS: Phosphate-buffered saline
PDX: Patient-derived xenograft
ROS: Reactive oxygen species
RT–PCR: Real-time reverse transcription–polymerase chain reaction
SABG: Senescence-associated β-galactosidase
SE: Standard error
ssDNA: Single-stranded DNA
TCA: Tricarboxylic acid
THC: Theracurmin^®^
8-OHdG: 8-hydroxy-2′-deoxyguanosine
