# Supplementary material for: Combination treatment with highly bioavailable curcumin and NQO1 inhibitor exhibits potent antitumor effects on esophageal squamous cell carcinoma
Source: J Gastroenterol. 2019 Feb 8;54(8):687–98. doi: 10.1007/s00535-019-01549-x (PMC6647399; doi:10.1007/s00535-019-01549-x)
Supplement: Supplementary file 1 — Supplementary material 1 (DOCX 26 kb) [file 535_2019_1549_MOESM1_ESM.docx]

**Supplementary materials and methods**

**Cell lines and cell culture**

We used human ESCC cell lines TE-1, TE-5, TE-6, TE-8, TE-10, and TE-11, which were obtained from Riken BioResource Center (Ibaraki, Japan), T.Tn, which was purchased from JCRB (Osaka, Japan), and HCE-4, which was provided by the Cell Culture Core of the University of Pennsylvania. TE-11R is 5-fluorouracil (FU)-resistant ESCC cells that have been described previously [1, 2]. The characteristics of these cells are summarized in **Supplementary Table 2** [3, 4]. HEK293T, a cell line derived from human embryonic kidney cells, was used to produce lentivirus, and was purchased from DS Pharma Biomedical Co., Ltd. (Osaka, Japan). Two independent immortalized normal human esophageal keratinocyte cell lines, EPC1-hTERT and EPC2-hTERT, were obtained from the Cell Culture Core of the Center for Molecular Studies in Digestive and Liver Diseases at the University of Pennsylvania [5, 6]. ESCC cells except HCE-4 were cultured in RPMI1640 medium (Life Technologies Corp., Grand Island, NY, USA). HCE-4 and HEK293T cells were cultured in DMEM medium (Life Technologies). These media were supplemented with 10% fetal bovine serum (Life Technologies), 100 μg/mL of streptomycin and 100 units/mL of penicillin (Life Technologies). EPC1-hTERT and EPC2-hTERT cells were cultured in Keratinocyte Serum-Free medium (Life Technologies), supplemented with 40 μg/mL of Bovine Pituitary Extract, 1 ng/mL Epidermal Growth Factor, 100 μg/mL of streptomycin, and 100 units/mL of penicillin (Life Technologies). All cells were cultured at 37 °C in a 5% CO_2_ incubator.

**WST-1 cell viability assay**

Cell viability was determined using the WST-1 assay (Roche Applied Science, Upper Bavaria, Germany) according to the manufacturer’s instructions. Cells (1–5 × 10^3^ cells/well) were seeded in 96-well plates and exposed to the indicated concentrations (0–50 μM) of THC (Theravalues Corporation, Tokyo, Japan) or curcumin (Nacalai tesque, Inc., Kyoto, Japan) for 96 h. All data were obtained with a multiwell plate reader (Infinite 200 Pro, Tecan, Männedorf, Switzerland) at wavelengths of 450 nm and 630 nm.

**Caspase-Glo^®^ 3/7 assay**

The caspase-3 and caspase-7 activities of ESCC cells were evaluated using the Caspase-Glo^®^ 3/7 Assay (Promega, Madison, WI, USA) according to the manufacturer’s instructions. Cells (2 × 10^3^ cells/well) were plated in 96-well plates. After 12-h (TE-5) or 24-h (TE-8 and TE-11R) treatment with THC (0–50 μM), the Caspase-Glo^®^ 3/7 reagent was added to each well, the plates were incubated for 45 min at 37 °C, then the luminescence was measured using a plate-reading luminometer (GloMax^®^-Multi Detection System, Promega).

**Spheroid assay**

Cells (100 cells/well) were seeded in 96-well ultra-low attachment plates (Corning, New York, NY, USA) in MEGM™ Mammary Epithelial Cell Growth Medium (Lonza, Basal, Switzerland) and cultured with the indicated concentrations (0–50 μM) of THC for 2 weeks. The CellTiter-Glo^®^ 3D Cell Viability reagent (Promega) was added to each well, and the plates were incubated for 30 min at room temperature, then the luminescence was measured using a plate-reading luminometer (GloMax^®^-Multi Detection System).

**Soft agar colony formation assay**

Anchorage-independent growth activities were determined using a soft agar colony formation assay. This assay was performed in 6-well plates with a base of 1.5 mL of medium containing 1% NuSieve™ GTG™ Agarose (Lonza). Cells (2.5 × 10^4^ cells/well) were seeded in 1.5 mL of medium containing 0.67% agar and layered onto the base, then 2 mL of medium was added on top of the agar gel. Colonies were stained with 0.02% Giemsa Stain Solution (Muto Pure Chemicals Co., Ltd., Tokyo, Japan) at day 14 (TE-11R) or day 21 (TE-8) and 15 photographs of colonies were taken for each density using a Keyence BIOREVO BZ-9000 microscope (Keyence Corp., Osaka, Japan). Colonies larger than 50 μm were counted in the phase-contrast images.

**Microarray hybridization**

Cells (5 × 10^5^ cells) were treated with 10 μM THC for 4 h, 8 h, or 24 h. Isolated RNA from each group was reverse transcribed, amplified, and labeled using the Low Input Quick Amp Labeling Kit (Agilent Technologies, Santa Clara, CA, USA). Quantification of total RNA was performed using a NanoDrop^®^ 2000 (ThermoFisher Scientific, Grand Island, NY, USA) and the quality of the total RNA was assessed using TapeStation 2200 (Agilent Technologies). Quantification and specific activity of labeled cRNA were determined using the NanoDrop^®^ 2000 (ThermoFisher Scientific). A total of 600 ng of cRNA was then applied to the SurePrint G3 Human Gene Expression v2 8 × 60 K Microarray Kit (#G4858A-039494, Agilent Technologies) (reference sequence genes targeted: 24588) as per the manufacturer’s instructions and hybridized in a rotating oven for 17 h at 65 °C. Arrays were washed and then scanned using a Microarray Scanner G2565CA (Agilent Technologies). Feature extraction software was utilized to extract gene expression data (Agilent Technologies). We used GPL16699 platform (Agilent-039494 SurePrintG3 Human GE v2 8 × 60K Microarray 039381, Feature Number version). The signal values were corrected using background signal and processed quantile normalization and log 2 transformation. After the filtering with the quality of signal, differential expression genes (DEGs) were detected using limma [7]. The comparisons were made between ESCC cells (TE5/TE8 without THC treatment, and TE5/TE8 after 4 h, 8 h and 24 h THC treatment) to investigate the differences of gene expression pattern after THC treatment. The detected DEGs for each comparison were filtered to those having a false discovery rate (FDR) < 0.05 and log2 fold changes > 2.

**Real-time reverse transcription–PCR**

RNA extraction, cDNA synthesis, and real-time reverse transcription–polymerase chain reaction (RT–PCR) was performed as described previously [4]. The relative expression of each mRNA was normalized to β-actin as an internal control. The primers used in this study were as follows: *KEAP1*: forward 5′-GGCCAAGCAAGAGGAGTTCT-3′; reverse 5′-GGGGTTGTAACAGTCCAGGG-3′; *NRF2*: forward 5′-GCTTGGAGGCTCATCTCACA-3′; reverse 5′-TGCCATCTCTTGTTTGCTGC-3′; *NMRAL2P*: forward 5′-CTGGAGAACGTCAAGCGACT-3′; reverse 5′-GGGATGCCAATGGACCAGAA-3′; *NQO1*: forward 5′-AAAGGACCCTTCCGGAGTAA-3′; reverse 5′-AGGCTGCTTGGAGCAAAATA-3′; *ACTB*: forward 5′-TTGTTACAGGAAGTCCCTTGCC-3′; reverse 5′-ATGCTATCACCTCCCCTGTGTG-3′.

**Western blotting**

Whole cell lysates were collected and western blotting was performed as described previously [4]. Primary antibodies and titers were as follows: rabbit polyclonal anti-PARP antibody (#9542, Cell Signaling Technology, Inc., Danvers, MA, USA; 1:1000), rabbit monoclonal anti-NRF2 antibody (#ab62352, Abcam Inc., Cambridge, MA, USA; 1:1000), mouse monoclonal anti-NQO1 antibody (A180, #3187, Cell Signaling; 1:1000), rabbit polyclonal anti-α-tubulin antibody (#ab4074, Abcam; 1:400), rabbit polyclonal anti-histone H1 antibody (FL-219, #sc-10806, Santa Cruz Biotechnology, Inc., Santa Cruz, CA, USA; 1:200), and rabbit monoclonal anti-β-actin antibody (13E5, #4970, Cell Signaling; 1:2000). The secondary antibodies and titers were HRP-linked whole Ab donkey anti-rabbit IgG, (#NA-934, GE Healthcare, Little Chalfont, UK; 1:2000) and HRP-linked whole Ab sheep anti-mouse IgG, (#NA-931, GE Healthcare; 1:2000).

**Chromatin protein isolation**

Chromatin protein isolation was performed as previously described [8]. Cells were treated with 10 μM THC for 24 h or siRNA for 48 h and resuspended (2 × 10^6^ cells/mL) in buffer A (10 mM HEPES [pH 7.9], 10 mM KCl, 1.5 mM MgCl_2_, 0.34 M sucrose, 10% glycerol, 1 mM DTT, 1 mM PMSF). Triton X-100 (0.1%) was added and samples centrifuged at 3500 *g*, 4 °C for 5 min. This supernatant was defined as the cytosolic fraction. The pellet was washed once in buffer A, and then lysed in buffer B (3 mM EDTA, 0.2 mM EGTA, 1 mM DTT, 1 mM PMSF). Insoluble chromatin was collected by centrifugation at 3500 *g*, 4 °C for 5 min, washed once in buffer B, and centrifuged again under the same conditions. The final chromatin pellet was resuspended in 2 × SDS–PAGE sample buffer (0.125 M Tris-HCl [pH 6.8], 20% glycerol, 4% SDS, 10% 2-mercaptoethanol) and was defined as the chromatin fraction. To perform western blotting, the cytosolic fraction was mixed with an equal volume of 2 × SDS–PAGE sample buffer.

**siRNA transfection**

Gene-speciﬁc siRNAs or a MISSION^®^ siRNA Universal Negative Control (Sigma–Aldrich, St. Louis, MO, USA) were transfected into cells using Lipofectamine^®^ RNAiMax transfection reagent (Invitrogen, Carlsbad, CA, USA), according to the manufacturer’s protocol. The sequences of the siRNAs used in this study were as follows: *KEAP1*: GUGUUACGACCCAGAUACA; *NRF2*: CAAACAGAAUGGUCCUAAA; *NMRAL2P*: GCCAAUAUGUGUCGUUUCU.

**Measurement of intracellular ROS levels**

The generation of intracellular reactive oxygen species was measured using an OxiSelect^TM^ Intracellular ROS assay kit (Cell Biolabs, Inc., San Diego, CA, USA). 2′,7′-Dichlorodihydrofluorescein diacetate (DCFH-DA) was added to cells 1 h after treatment with THC (0–20 μM), curcumin(0–20 μM) and/or ES936 (50 nM), and intracellular ROS levels were quantified by detecting oxidized fluorescent 2′,7′-dichlorodihydrofluorescein (DCF) using a multiwell fluorometric plate reader (Infinite 200 Pro) at 485/535 nm.

**Measurement of mitochondrial ROS levels**

The generation of mitochondrial ROS was measured using an OxiORANGE^TM^ Mitochondrial ROS assay kit (GORYO Chemical, Inc., Sapporo, Japan). An oxidation-sensitive probe was added, and then cells were treated with THC (0–20 μM) for 1 h. Mitochondrial ROS levels were quantified by detecting oxidized fluorescent probe using a multiwell fluorometric plate reader (Infinite 200 Pro) at 535/590 nm.

**Immunofluorescent staining for 8-OHdG**

DNA oxidation was assessed by 8-OHdG [9]. The cells were fixed in 4% paraformaldehyde and treated with RNase (100 mg/mL) in Tris buffer (10 mM Tris-HCl [pH 7.5], 1 mM EDTA, and 0.4 M NaCl) at 37 °C for 1 h. After rinsing with phosphate-buffered saline (PBS), cells were treated with proteinase K (10 mg/mL) at room temperature for 7 min. After another rinse with PBS, DNA was denatured by treatment with 4 N HCl for 7 min at room temperature. The pH was adjusted with 50 mM Tris-HCl for 5 min at room temperature. Cells were rinsed with 0.2% Triton X and 0.5% goat serum in PBS, and stained with a monoclonal anti-8-OHdG antibody (15A3, #sc-66036, Santa Cruz; 1:50). Alexa Fluor 594-conjugated goat anti-mouse IgG (#8890, Cell Signaling; 1:500) was used as the secondary antibody. The coverslips were mounted with Vectashield mounting solution including DAPI (Vector Labs, CA, USA). Fluorescence images were captured and the fluorescent intensity of 8-OHdG from approximately 350 cells was measured using a Keyence BIOREVO BZ-9000 microscope (Keyence Corp., Osaka, Japan).

**Cell cycle assay**

Cells (2.5 × 10^4^ cells/well) were seeded in 24-well plates and exposed to the indicated concentrations (0–50 μM) of THC for 24 h. The Cell-Clock Cell Cycle Assay kit (Biocolor Life Science Assays, Antrim, UK) was used to stain cells, which were scored by counting at least 100 cells high-power field (n = 6) under light microscopy.

**Senescence-associated β-galactosidase (SABG) assay**

Cells (1 × 10^5^ cells/well) were seeded in 6-well plates and exposed to the indicated concentrations (0–50 μM) of THC for 24 h. The Senescence β-Galactosidase Staining kit (Cell Signaling) was used to stain senescent cells, which were scored by counting at least 100 cells high-power field (n = 6) under light microscopy.

**Metabolite analysis**

Metabolite analysis was performed using C-SCOPE (Human Metabolome Technologies, Yamagata, Japan), according to the recommended protocol [10]. Cells (5 × 10^5^ cells) were treated with 10 μM THC for 8 h, washed twice with 5% mannitol solution, and treated with methanol and 4 μM internal standard. After centrifugation at 2300 *g*, 4 °C for 5 min, the supernatants were collected for centrifugal filtration through a 5-kDa-cutoff filter at 9100 *g*, 4 °C for 3 h. Concentrations of all charged compounds were measured by capillary electrophoresis time-of-flight mass spectrometry and capillary electrophoresis tandem mass spectrometry as described previously [10-12].

**Vectors and viral infections**

The following lentiviral vectors were used in this study: GIPZ nonsilencing Lentiviral shRNA control (control for *NQO1* knockdown), V3LHS_331229 (shNQO1-1), V3LHS_331230 (shNQO1-2), V3LHS_331231 (shNQO1-3), LentiORF control (control for *NQO1* overexpression), and PLOHS_100005522 (NQO1 overexpression) (GE Healthcare Dharmacon, Lafayette, CO). They were transfected into HEK293T to produce viral particles using the Trans-Lentiviral shRNA Packaging Kit (GE Healthcare Dharmacon). TE-11R cells were incubated with RPMI1640 medium containing these viral particles and 10 μg/mL hexamethrine bromide, then, centrifuged at 1800 rpm, 32 °C for 1 h. After centrifugation, the medium was replaced with fresh RPMI1640 medium containing viral particles and 10 μg/mL hexamethrine bromide, and centrifuged again under the same conditions. After centrifugation, the medium was changed to normal RPMI1640. 1.5 μg/mL puromycin (knockdown) or 20 μg/mL Blasticidin S (overexpression) were used for cell selection.

**Immunohistochemical staining**

Mice were painlessly sacrificed under appropriate anaesthesia with the inhalation of isoflurane (Escain, Mylan Pharmaceuticals, Tokyo, Japan) and cervical dislocation, and tissue samples were collected in 10% formaldehyde phosphate buffer solution (Wako Pure Chemical Industries, Ltd.). Incubation and washing procedures were carried out at room temperature. After deparaffinization and antigen retrieval, endogeneous peroxidase activity was blocked by 0.3% H_2_O_2_ in methyl alcohol for 30 min (Ki67, ssDNA, NRF2 and NQO1). The glass slides were washed in PBS (6 times, 5 each min) and mounted with 1% horse normal serum in PBS for 30 min. Subsequently primary antibody was applied overnight at 4 °C. They were incubated with biotinylated horse anti-mouse serum (second antibody) diluted to 1:300 in PBS for 40 min, followed by washes in PBS (6 times, 5 min). Avidin-biotin-peroxidase complex (Ki67, ssDNA, NRF2 and NQO1) or avidin-biotin-alkaliphosphatase complex (8-OHdG) (Vector Laboratories, Burlingame, CA) at a dilution of 1:100 in BSA was applied for 50 min. After washing in PBS (6 times, 5 min), coloring reaction was carried out with DAB (Ki67, ssDNA, NRF2 and NQO1) or Fast red (8-OHdG), and nuclei were counterstained with hematoxylin.

Primary antibodies and titers were as follows: mouse anti-Ki67 antibody (#NCL-L-Ki67-MM1, Leica Biosystems, Nussloch, Germany; 1:100) ,rabbit anti-single stranded DNA antibody (#18731, Immuno-Biological Laboratories Co., Ltd., IBL., Tochigi, Japan; 1:600), rabbit polyclonal anti-NRF2 antibody (#GTX103322, GeneTex, LA, USA; 1:200), goat polyclonal anti-NQO1 antibody (#ab2346, Abcam; 1:200), and mouse anti-8-OHdG antibody (N45.1, #MOG-020P, Japan Institute for the Control of Aging, NIKKEN SEIL Co., Ltd, JalCA, Shizuoka, Japan; 10 μg/ml).

**References**

1. Ohashi S, Kikuchi O, Tsurumaki M, et al. Preclinical validation of talaporfin sodium-mediated photodynamic therapy for esophageal squamous cell carcinoma. PLoS One. 2014;9:e103126.

2. Kikuchi O, Ohashi S, Nakai Y, et al. Novel 5-fluorouracil-resistant human esophageal squamous cell carcinoma cells with dihydropyrimidine dehydrogenase overexpression. Am J Cancer Res. 2015;5:2431–40.

3. Nishihira T, Hashimoto Y, Katayama M, et al. Molecular and cellular features of esophageal cancer cells. J Cancer Res Clin Oncol. 1993;119:441–9.

4. Yoshioka M, Ohashi S, Ida T, et al. Distinct effects of EGFR inhibitors on epithelial- and mesenchymal-like esophageal squamous cell carcinoma cells. J Exp Clin Cancer Res. 2017;36:101.

5. Harada H, Nakagawa H, Oyama K, et al. Telomerase induces immortalization of human esophageal keratinocytes without p16INK4a inactivation. Molecular cancer research : MCR. 2003;1:729–38.

6. Ohashi S, Natsuizaka M, Yashiro-Ohtani Y, et al. NOTCH1 and NOTCH3 coordinate esophageal squamous differentiation through a CSL-dependent transcriptional network. Gastroenterology. 2010;139:2113–23.

7. Ritchie ME, Phipson B, Wu D, et al. limma powers differential expression analyses for RNA-sequencing and microarray studies. Nucleic Acids Res. 2015;43:e47.

8. Mendez J, Stillman B. Chromatin association of human origin recognition complex, cdc6, and minichromosome maintenance proteins during the cell cycle: assembly of prereplication complexes in late mitosis. Mol Cell Biol. 2000;20:8602–12.

9. Cooke MS, Evans MD, Burd RM, et al. Induction and excretion of ultraviolet-induced 8-oxo-2'-deoxyguanosine and thymine dimers in vivo: implications for PUVA. J Invest Dermatol. 2001;116:281–5.

10. Makinoshima H, Takita M, Matsumoto S, et al. Epidermal growth factor receptor (EGFR) signaling regulates global metabolic pathways in EGFR-mutated lung adenocarcinoma. J Biol Chem. 2014;289:20813–23.

11. Soga T, Baran R, Suematsu M, et al. Differential metabolomics reveals ophthalmic acid as an oxidative stress biomarker indicating hepatic glutathione consumption. J Biol Chem. 2006;281:16768–76.

12. Sun L, Moritake T, Ito K, et al. Metabolic analysis of radioresistant medulloblastoma stem-like clones and potential therapeutic targets. PLoS One. 2017;12:e0176162.
