# Supplementary material for: Combination treatment with highly bioavailable curcumin and NQO1 inhibitor exhibits potent antitumor effects on esophageal squamous cell carcinoma
Source: J Gastroenterol. 2019 Feb 8;54(8):687–98. doi: 10.1007/s00535-019-01549-x (PMC6647399; doi:10.1007/s00535-019-01549-x)
Supplement: Supplementary file 2 — Supplementary material 2 (DOCX 14 kb) [file 535_2019_1549_MOESM2_ESM.docx]

**Supplementary Table 1. The IC_50_ values of Theracurmin^®^ on ESCC cells.**

| **Cell line** | **IC_50_ (μM)** |
| --- | --- |
| **TE-1**  **TE-5**  **TE-6**  **TE-8**  **TE-10**  **TE-11**  **TE-11R**  **T. Tn**  **HCE-4** | **19.23**  **19.45**  **7.03**  **8.88**  **12.91**  **8.98**  **34.98**  **19.66**  **8.94** |

The IC_50_ values of Theracurmin^®^ on each ESCC cell line are calculated from the cell survival shown in Fig. 1a and Supplementary Fig.1.
