# Supplementary material for: Combination treatment with highly bioavailable curcumin and NQO1 inhibitor exhibits potent antitumor effects on esophageal squamous cell carcinoma
Source: J Gastroenterol. 2019 Feb 8;54(8):687–98. doi: 10.1007/s00535-019-01549-x (PMC6647399; doi:10.1007/s00535-019-01549-x)
Supplement: Supplementary file 3 — Supplementary material 3 (DOCX 14 kb) [file 535_2019_1549_MOESM3_ESM.docx]

**Supplementary Table 2. The characteristics of the ESCC cells used in the current study.**

| **Cell** | **Differentiation** | **Phenotype** | **5-FU resistant** |
| --- | --- | --- | --- |
| **TE-1**  **TE-5**  **TE-6**  **TE-8**  **TE-10**  **TE-11**  **TE-11R**  **T.Tn**  **HCE-4** | **well**  **poor**  **well**  **moderate**  **well**  **moderate**  **moderate**  **–**  **–** | **epithelial**  **epithelial**  **epithelial**  **mesenchymal**  **epithelial**  **epithelial**  **epithelial**  **epithelial**  **mesenchymal** | **no**  **no**  **no**  **no**  **no**  **no**  **yes**  **no**  **no** |

Cellular characteristics in terms of differentiation, phenotypes, and 5-FU resistance are summarized. poor: poorly differentiated, moderate: moderately differentiated, well: well differentiated, –: unknown.
