# Supplementary material for: Combination treatment with highly bioavailable curcumin and NQO1 inhibitor exhibits potent antitumor effects on esophageal squamous cell carcinoma
Source: J Gastroenterol. 2019 Feb 8;54(8):687–98. doi: 10.1007/s00535-019-01549-x (PMC6647399; doi:10.1007/s00535-019-01549-x)
Supplement: Supplementary file 4 — Supplementary material 4 (DOCX 20 kb) [file 535_2019_1549_MOESM4_ESM.docx]

**Supplementary Figure Legends**

**Supplementary Fig. 1**

**Cytotoxic effects of Theracurmin^®^ against ESCC cells.** Cell viability: ESCC cells were cultured with the indicated concentrations (0–50 μM) of Theracurmin^®^ (THC) for 96 h. Cell viability was measured using the WST-1 assay. Viability of each cell type treated with THC relative to that of untreated cells is indicated. (**P <* 0.05, ***P <* 0.01, *vs* untreated cells; n = 3)

**Supplementary Fig. 2**

**Effect of Theracurmin^®^ on cell cycle and senescence.** (a) Cell cycle: ESCC cells (TE-5, TE-8, and TE-11R cells) were cultured with the indicated concentrations (0–50 μM) of Theracurmin^®^ (THC) for 24 h. Cell cycle was measured using the cell-clock cell cycle assay. Cells per high-power field (HPF) were counted in 6 random fields. (b) Cell senescence: ESCC cells (TE-8 cells) were cultured with the indicated concentrations (0–50 μM) of THC for 24 h. Cell senescence was assessed with senescence-associated β-galactosidase (SABG) assay. Cells per HPF were counted in 6 random fields. (**P <* 0.05, ***P <* 0.01, *vs* untreated cells)

**Supplementary Fig. 3**

**Upregulation of NMRAL2P after Theracurmin^®^ treatment.** Genes upregulated in Theracurmin^®^ (THC)-treated ESCC cells: Microarray gene expression analysis identified several genes that were upregulated in ESCC cells (TE-5 and TE-8 cells) treated with THC (10 μM) for 4 h, 8 h, or 24 h.

**Supplementary Fig. 4**

**Activation of NRF2–NMRAL2P–NQO1 pathway by THC.** (a) KEAP1 mRNA levels in *KEAP1* knockdown cells treated with or without Theracurmin^®^ (THC): RT–PCR showed efficient *KEAP1* knockdown using siRNA in TE-5 and TE-8 cells. KEAP1 mRNA level of each cell type relative to that of control cells untreated with THC is indicated. β-actin served as an internal control. (**P <* 0.05, ***P <* 0.01, *vs* the control cells; n = 3) (b) NRF2 mRNA levels in *NRF2* knockdown cells treated with or without THC: RT–PCR showed efficient *NRF2* knockdown using siRNA in TE-5 and TE-8 cells. NRF2 mRNA level of each cell type relative to that of control cells untreated with THC is indicated. β-actin served as an internal control. (**P <* 0.05, *vs* the control cells; n = 3). (c) NRF2 protein levels in *NRF2* knockdown cells: Cellular protein was divided into cytosolic fraction (Cy) and chromatin fraction (Ch). Western blotting showed efficient nuclear *NRF2* knockdown using siRNA in TE-5 and TE-8 cells. α-Tubulin or histone H1 served as a loading control for cytosolic or chromatin extracts, respectively. (d) NMRAL2P expression levels in *NMRAL2P* knockdown cells treated with or without THC: RT–PCR showed efficient *NMRAL2P* knockdown using siRNA in TE-5 and TE-8 cells. NMRAL2P level of each cell type relative to that of control cells untreated with THC is indicated. β-actin served as an internal control. (**P <* 0.05, ***P <* 0.01, *vs* the control cells; n = 3). (e) NMRAL2P expression levels in *KEAP1* or *NRF2* knockdown cells treated with or without THC: RT–PCR showed that siRNA-mediated knockdown of *KEAP1* and *NRF2* changed the expression level of NMRAL2P. NMRAL2P level of each cell type relative to that of control cells untreated with THC is indicated. β-actin served as an internal control. (**P <* 0.05, ***P <* 0.01, *vs* the control cells; n = 3). (f) NQO1 mRNA levels in *NMRAL2P* knockdown cells treated with or without THC: RT–PCR showed that siRNA-mediated knockdown of *NMRAL2P* changed the mRNA levels of NQO1 in TE-5 and TE-8 cells. NQO1 mRNA level of each cell type relative to that of control cells untreated with THC is indicated. β-actin served as an internal control. (**P <* 0.05, *vs* the control cells; n = 3). (g) NQO1 protein levels in *NMRAL2P*, *NRF2*, or *KEAP1* knockdown cells treated with or without THC: Western blotting showed that siRNA-mediated knockdown of *NMRAL2P*, *NRF2*, and *KEAP1* changed the protein level of NQO1 in TE-5 and TE-8 cells. β-actin served as a loading control. THC: treatment with THC (10 μM) for 24 h.

**Supplementary Fig. 5**

**The levels of KEAP1 mRNA, nuclear NRF2 protein, and NQO1 protein in ESCC cells under the basal-line condition.** (a) KEAP1 mRNA levels: RT–PCR showed KEAP1 mRNA levels in immortalized normal esophageal epithelial cells (EPC1-hTERT and EPC2-hTERT cells) and ESCC cells (TE1, TE5, TE6, TE8, TE10, TE11, TE11R, T.Tn, and HCE4 cells) under the base-line condition. KEAP1 mRNA level of each cell type relative to that of EPC1-hTERT is indicated. β-actin served as an internal control. (n = 3) (b) NRF2 protein levels in chromatin fraction: Western blotting with protein of chromatin fraction showed NQO1 protein levels in immortalized normal esophageal epithelial cells and ESCC cells under the base-line condition. Histone H1 served as a loading control for chromatin extracts. Densitometry is the relative intensities of NRF2 after normalization for the levels of histone H1. (c) NQO1 protein levels: Western blotting showed NQO1 protein levels in immortalized normal esophageal epithelial cells and ESCC cells under the base-line condition. β-actin served as a loading control.

**Supplementary Fig. 6**

**Cytotoxic effects of curcumin on ESCC cells.** (a) ROS production: ESCC cells were cultured with the indicated concentrations (0–20 μM) of curcumin for 1 h. Intracellular ROS levels were measured using the DCF assay. Relative DCF fluorescence intensity of each cell treated with the indicated concentrations of curcumin relative to that of untreated cells is indicated. (***P <* 0.01, *vs* untreated cells; n = 3). (b) Cell viability: ESCC cells were cultured with the indicated concentrations (0–50 μM) of curcumin for 96 h. Cell viability was measured using the WST-1 assay. Viability of each cell type treated with curcumin relative to that of untreated cells is indicated. (**P <* 0.05, ***P <* 0.01, *vs* untreated cells; n = 3). (c) Assessment of apoptosis: TE-5 cells were cultured with the indicated concentrations (0–20 μM) of curcumin and ES936 (50 nM) for 24 h. The cleavage of PARP was investigated by western blotting. β-actin served as a loading control.

**Supplementary Fig. 7**

**Working model of Theracurmin^®^ effects on ESCC cells.** Theracurmin^®^ (THC) induces ROS and mitochondrial damage, which leads to cytotoxic and antitumor effects in ESCC cells. Here, THC upregulates the NRF2–NMRAL2P–NQO1 pathway, which attenuates ROS production. NQO1 inhibitor can reinforce the antitumor effects of THC.
