# Supplementary figures and images for: Combination treatment with highly bioavailable curcumin and NQO1 inhibitor exhibits potent antitumor effects on esophageal squamous cell carcinoma
Source: J Gastroenterol. 2019 Feb 8;54(8):687–98. doi: 10.1007/s00535-019-01549-x (PMC6647399; doi:10.1007/s00535-019-01549-x)

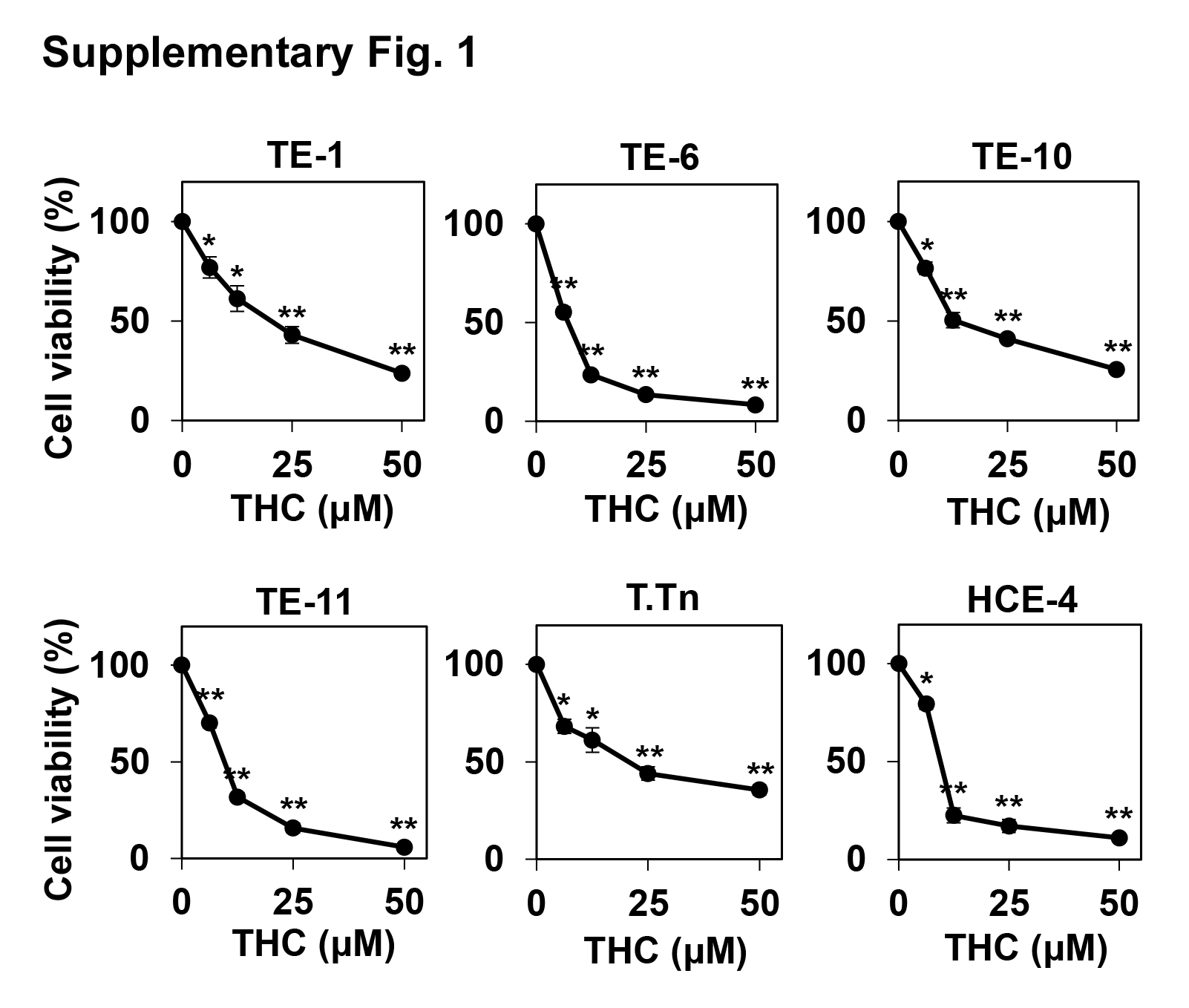

Supplement: Supplementary file 5 — Supplementary material 5 (TIFF 1933 kb) [file 535_2019_1549_MOESM5_ESM.tif]

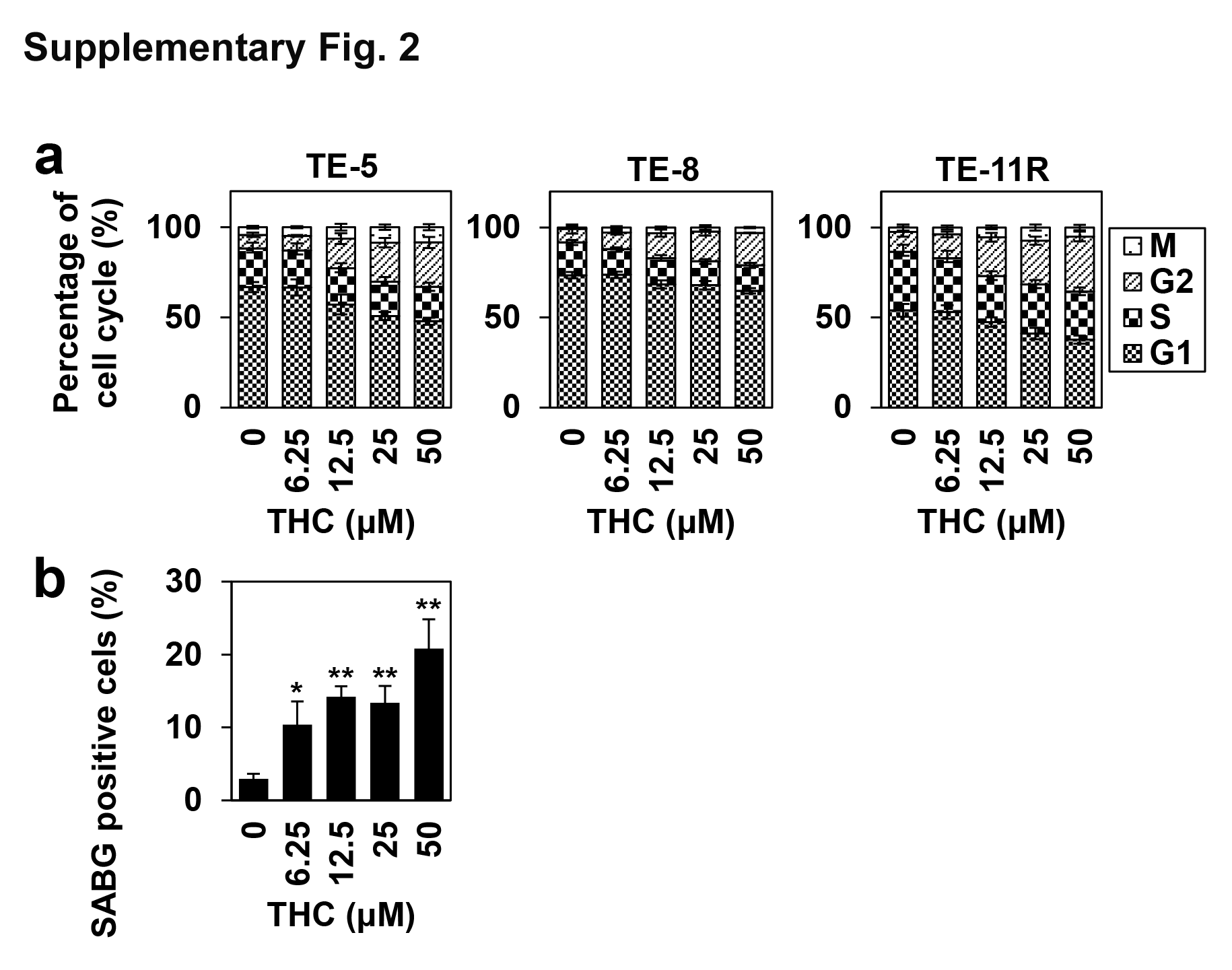

Supplement: Supplementary file 6 — Supplementary material 6 (TIFF 2282 kb) [file 535_2019_1549_MOESM6_ESM.tif]

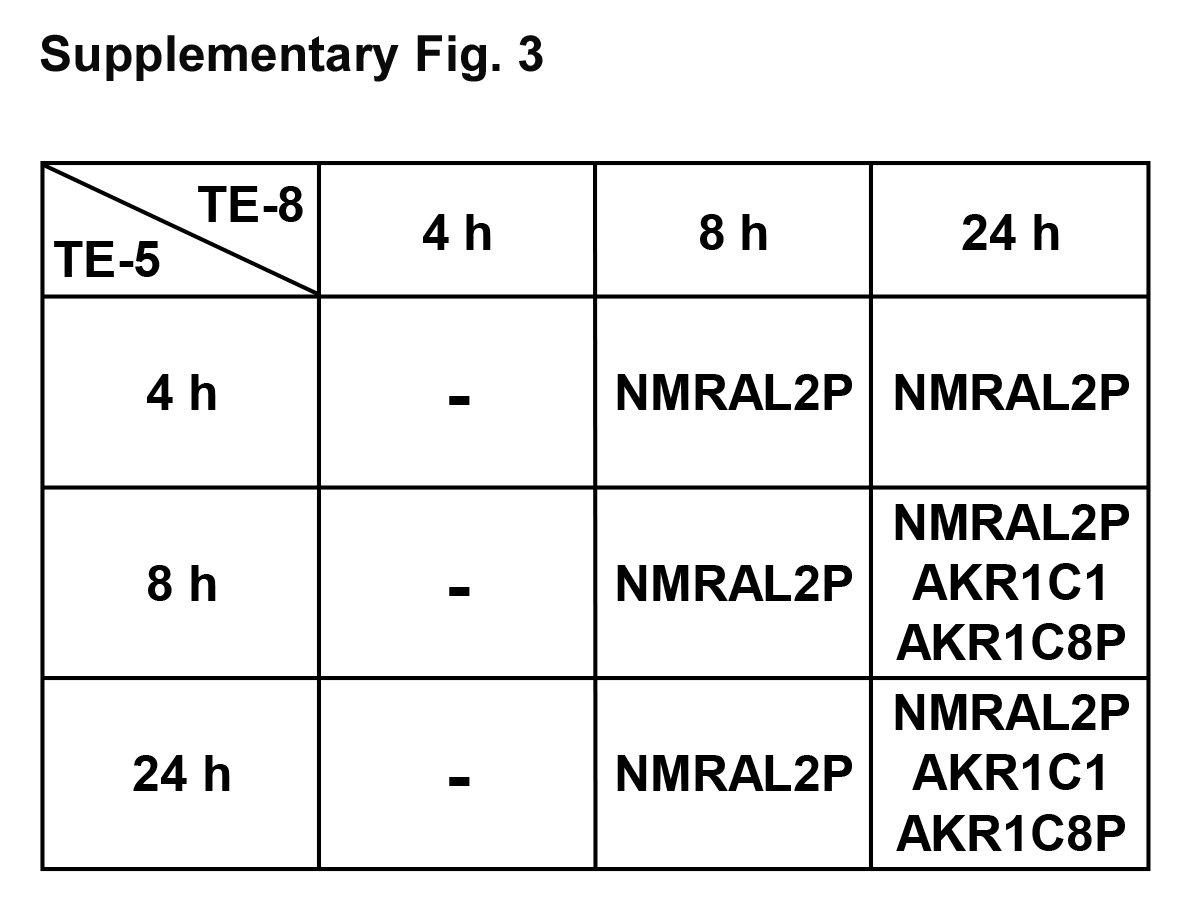

Supplement: Supplementary file 7 — Supplementary material 7 (TIFF 936 kb) [file 535_2019_1549_MOESM7_ESM.tif]

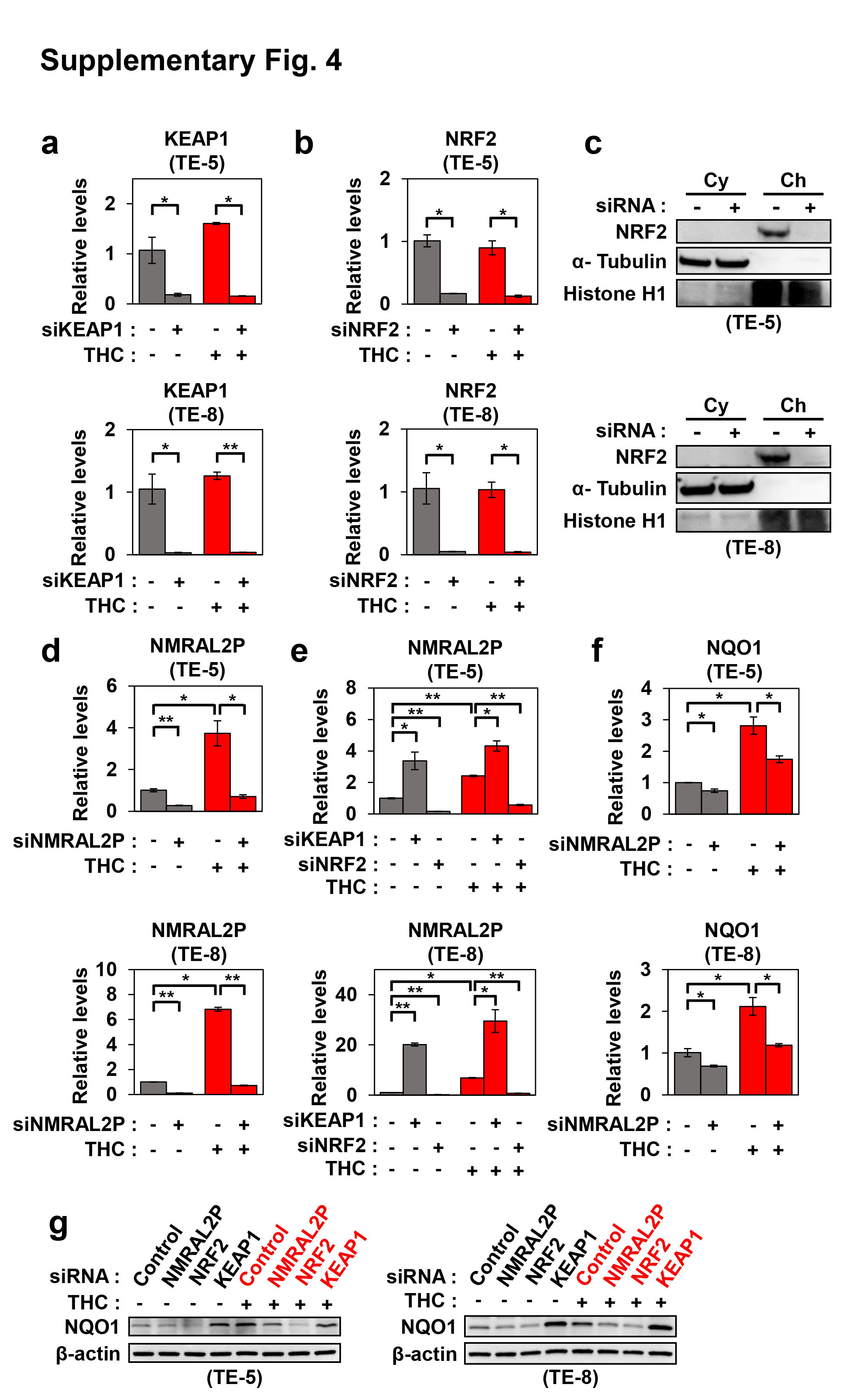

Supplement: Supplementary file 8 — Supplementary material 8 (TIFF 571 kb) [file 535_2019_1549_MOESM8_ESM.tif]

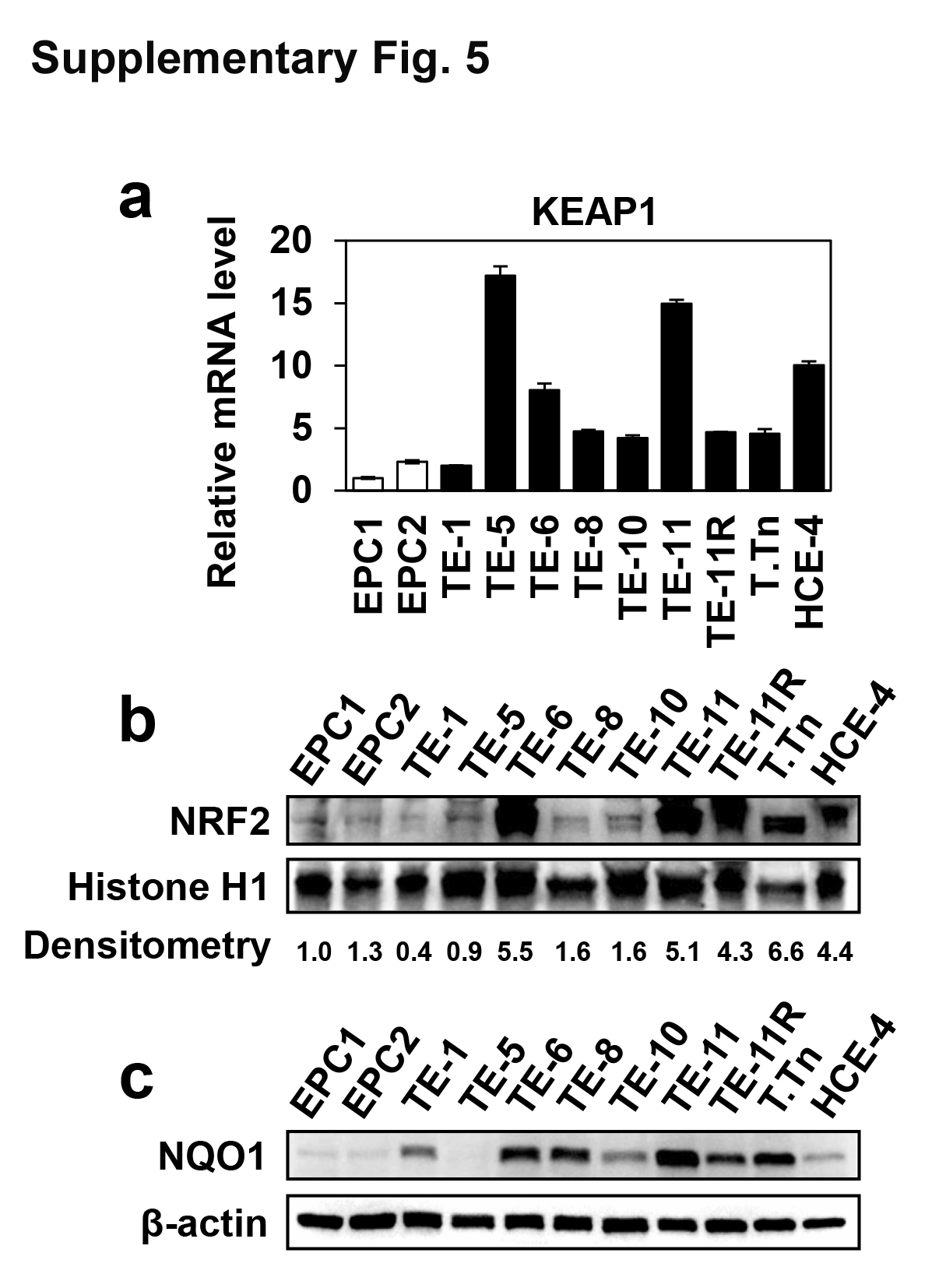

Supplement: Supplementary file 9 — Supplementary material 9 (TIFF 1715 kb) [file 535_2019_1549_MOESM9_ESM.tif]

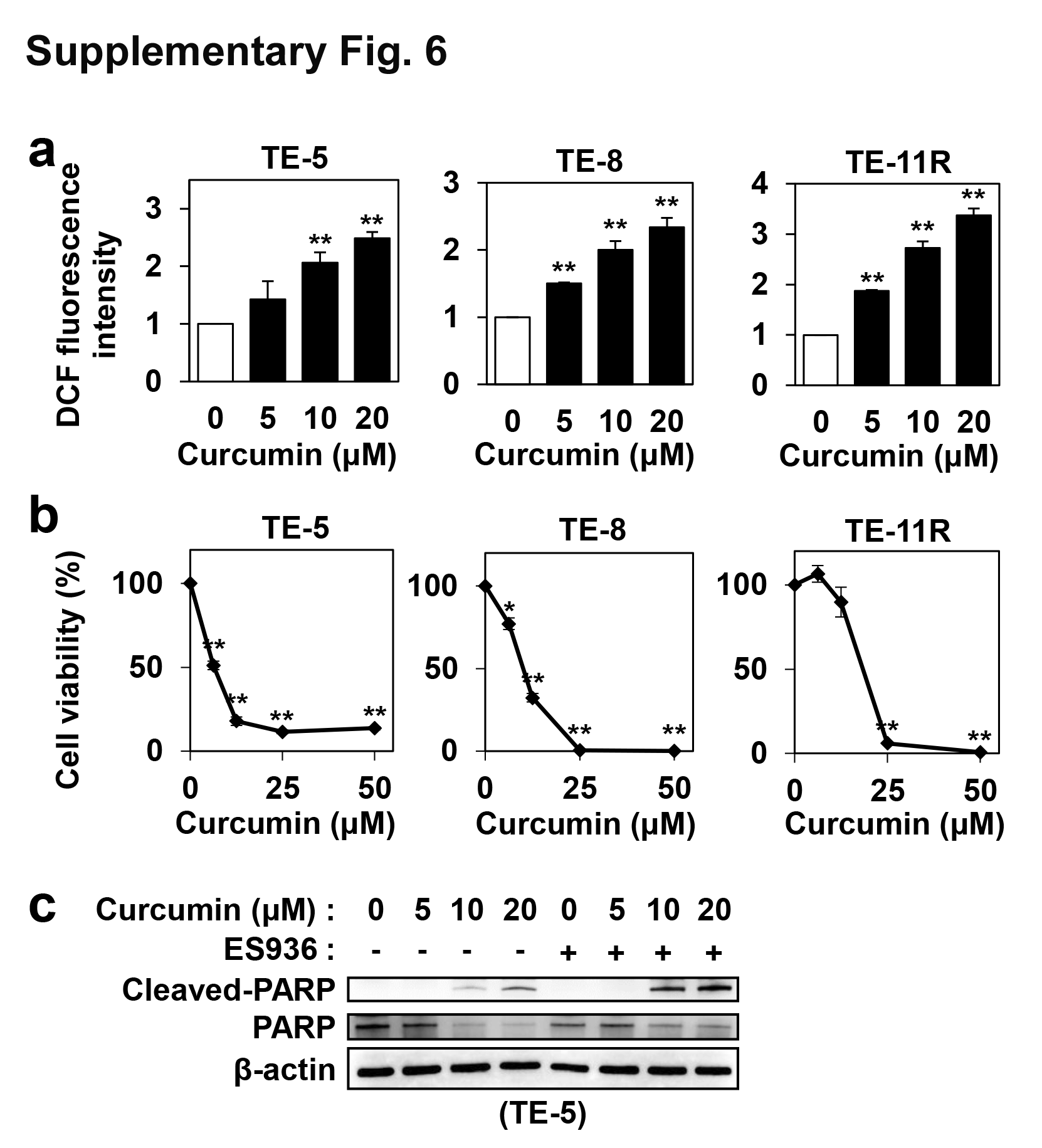

Supplement: Supplementary file 10 — Supplementary material 10 (TIFF 2676 kb) [file 535_2019_1549_MOESM10_ESM.tif]

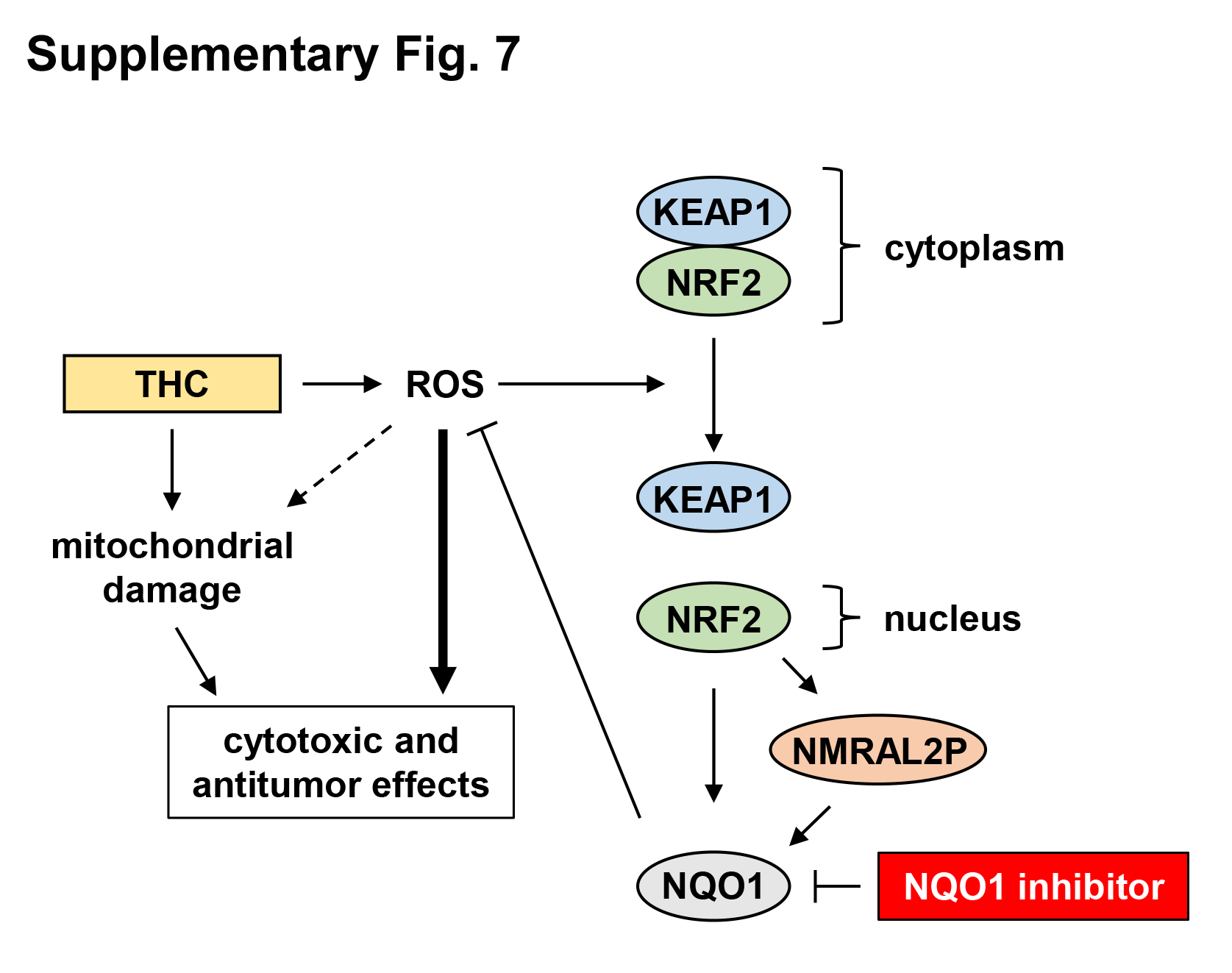

Supplement: Supplementary file 11 — Supplementary material 11 (TIFF 138 kb) [file 535_2019_1549_MOESM11_ESM.tif]
